# Supplementary material for: Distinct activation mechanisms regulate subtype selectivity of Cannabinoid receptors
Source: Commun Biol. 2023 May 5;6:485. doi: 10.1038/s42003-023-04868-1 (PMC10163236; doi:10.1038/s42003-023-04868-1)
Supplement: Supplementary file 1 — Supporting Information [file 42003_2023_4868_MOESM1_ESM.pdf]

Supporting information: Distinct Activation Mechanisms Regulate Subtype Selectivity of Cannabinoid Receptors

Soumajit Dutta[1] and Diwakar Shukla[*,1,2,3]

1*Department of Chemical and Biomolecular Engineering, University of Illinois at Urbana-Champaign, Urbana, IL, USA*

2*Center for Biophysics and Quantitative Biology, University of Illinois at Urbana-Champaign, Urbana, IL, USA*

3*Department of Bioengineering, University of Illinois at Urbana-Champaign, Urbana, IL, USA*

E-mail: diwakar@illinois.edu

List of Tables

1 PDB IDs with different ligands and downstream proteins S7

2 Simulation system details S8

3 Simulation details S9

4 Mean and standard deviation of structurally important features for every metastable state of CB_1 . . . . S10

5 Mean and standard deviation of structurally important features for every metastable state of CB_2 . . . . S11

6 Mean and standard error of the calculated ligand RMSD distribution for CB_1 (Intermediate state 2 and Active state) and CB_2 (Inactive and active state) . S15

7 Contact frequencies and corresponding interaction energies between ligand and protein residues for CB_2 . . . . S16

8 Distance features used to build Markov state model for CB_1 using RRCS. . . S17

9 Distance features used to build Markov state model for CB_2 using RRCS. . . S20

10 Distance and angle features used for adaptive sampling of CB_1. . . . . S52

11 Distance and angle features used for adaptive sampling of CB_2. . . . . S53

List of Figures

1 Binding pocket characterization of cannabinoid receptors S22

2 Indicators for characterizing important domain motions S23

3 Comparisons of conserved and non-conserved changes in apo and holo simulations S24

4 Conditional probabilities between structurally important features of CB_1 . . S25

5 MSM weighted 2-D free energy landscapes for structurally important features of CB_1 . . . . S26

6 Conditional probabilities between structurally important features of CB_2 . . S27

7 MSM weighted 2-D free energy landscapes for structurally important features of CB_2 . . . . . S28
8 Implied time-scale obtained using VAMPnets for CB_1 . . . . . S29
9 Implied time-scale obtained using VAMPnets for CB_2 . . . . . S30
10 State populations obtained using VAMPnets for CB_1 . . . . . S31
11 State populations obtained using VAMPnets for CB_2 . . . . . S32
12 Distributions of structurally important features for each metastable state of CB_2 . . . . . S33
13 MSM eigenvectors and tIC analysis for CB_1 . . . . . S34
14 Structural comparison of existing PDB with I2 metastable state S35
15 Distributions of structurally important features for each metastable state of CB_2 . . . . . S36
16 MSM eigenvectors and tIC analysis for CB_2 . . . . . S37
17 Kinetic monte carlo simulation S38
18 Backbone RMSD from the long timescale MD simulation performed on each metastable state S39
19 tIC analysis on long timescale MD simulation performed on each metastable state S40
20 RMSF from the long timescale MD simulation performed on each metastable state S41
21 Allosteric communication paths calculated for each metastable state for CB_1 S42
22 Allosteric communication paths calculated for each metastable state for CB_2 S43
23 CB_2 selective ligands S44
24 Pocket characteristics of I2 and active metastable states S45
25 Time series plots of ligand RMSDs from the long timescale MD simulation performed on ligand bound CB_1(intermediate state 2 and active state) and CB_2 (inactive and active states) metastable states S46

26 Density plot of ligand RMSDs from the long timescale MD simulation performed on ligand bound CB_1(intermediate state 2 and active state) and CB_2 (inactive and active states) metastable states S47
27 Contact frequencies between ligand and protein residues from long timescale MD simulation S48
28 Interaction energies between ligand and protein residues from long timescale MD simulation S49
29 Difference in ligand and protein contact frequencies between CB_1 and CB_2 . S50
30 Residue pair distances obtained from RRCS S51
31 Implied timescales and VAMP-2 scores obtained from MSM S54
32 Chapman–Kolmogorov test for CB_1 and CB_2 . . . . S55
33 Probability of populations Raw and MSM weighted states S55
34 Number of simulations from each state S56
35 Residues considered as docking center S56

Supplementary Note 1. Choice of the indicators of motion for different domains

The Domains' motions are classified as conserved and non-conserved motions (Figure 3). The non-conserved motion indicators were obtained from the discussion in the CB_1 and CB_2 structure papers backed by multiple structures of same conformational state. The conserved motion indicators have been discussed in many class A GPCR papers.[1]

Non-conserved Motions

N-terminus motion

Difference of the N-terminus position of CB_1 inactive and active structures have been discussed in X-ray crystal and Cryo-EM structure papers. As position of N-terminus changes from inside of the orthosteric pocket to the outside of the pocket, we selected the distance between N-terminus and conserved residue ($D^{2.50}$) close to the orthosteric binding pocket to reflect this motion. The $D^{2.50}$ is highly conserved across most of class A GPCRs and no major movement of this residue have been reported in the literature. Therefore, distance calculation from this residue will give proper view of N-terminus motion. We have used this distance in our previously published paper.[2]

Extracellular TM1 motion

Extracellular TM1 motion is also discussed in CB_1 X-ray crystal and Cryo-EM structure papers.[3,4] Therefore, to indicate this motion, we have used a residue in extracellular TM6, where no significant motion was observed in the crystal structure.

Toggle switch motions

The rotational movement of toggle switch ($W^{6.48}$ and $F^{3.36}$) residues has also been discussed in cannabinoid receptor structure papers and in context of other class A GPCRs.[4,5] We also

talked about the importance of this rotational movement in our previous paper.[2]

Hua *et al.* talked about toggle movement between the $W^{6.48}$ and $F^{3.36}$ during the activation of the receptors, where the pi-pi interaction between the two residues break during the activation.[3,4] These motions cause these residues' position to switch in z direction. Hence, this z direction motion was chosen as an indicator.

Intracellular TM5 motions

Hua *et al.* talked about side chain movement of $Y^{5.58}$ in the CB_2 towards the membrane.[6] Hence, we selected a residue in TM2 that is relatively stable to calculate the distance.

Conserved Motions

The conserved motions are observed across all class A GPCRs during the activations.[7–9] For example, intracellular TM6 was shown to move away from TM3 and intracellular TM7 was shown to move closer to TM5 during activation.[4] Thus, the indicators of these motions of intracellular TM6 and TM7 are chosen from the residues that are in TM3 and TM5, where domain motion is minimal. The indicators help to capture the difference between inactive and active experimentally determined structures of CB_1 and CB_2 (Figure 3).

Supplementary Table 1: CB_1 and CB_2 X-ray crystal and cryo-EM structures bound with different ligands and downstream proteins

	Antagonist	Agonist	Agonist and NAM	Agonist and G_i
CB_1	5TGZ (Inactive)[3]<br>5U09 (Inactive)[12]	5XRA (Active)[4]<br>5XR8 (Active)[4]<br>7V3Z (Active)[13]	6KQI (Inactive)[10]	6N4B (Active)[11]<br>6KPG (Active)[6]
CB_2	5ZTY (Inactive)[14]	6KPC (Inactive)[6]		6KPF (Active)[6]<br>6PT0 (Active)[15]

Supplementary Table 2: System details of apo and holo unbiased simulation of CB_1 and CB_2.

System	Simulation box size	Number of atoms	Number of Water molecules	Salt Concentration (mM)	Lipid Composition
CB_1 apo (Active)	$72 \times 72 \times 111$	51938	10798	150	POPC
CB_1 apo (Inactive)	$72 \times 72 \times 107$	48998	9866	150	POPC
CB_2 apo (Active)	$70 \times 70 \times 101$	45980	6650	150	POPC
CB_2 apo (Inactive)	$72 \times 72 \times 106$	49792	10166	150	POPC
CB_1 holo (Active)	$72 \times 72 \times 107$	51080	10451	150	POPC
CB_1 holo (Inactive)	$72 \times 72 \times 105$	46774	9119	150	POPC
CB_2 holo (Active)	$70 \times 70 \times 101$	45913	8827	150	POPC
CB_2 holo (Inactive)	$72 \times 72 \times 106$	47276	9320	150	POPC

Supplementary Table 3: Details of apo and holo (agonist and antagonist) unbiased simulation of CB_1 and CB_2.

Protein	Presence of Ligands	Total Simulation time (μs)
CB_1	apo (without ligand)	$\sim$ 419
CB_1	holo (with agonist bound)	$\sim$ 24
CB_1	holo (with antagonist bound)	$\sim$ 23
CB_2	apo (without ligand)	$\sim$ 278
CB_2	holo (with agonist bound)	$\sim$ 19
CB_2	holo (with antagonist bound)	$\sim$ 24

Supplementary Table 4: Mean and standard deviation of structurally important features for every metastable state of CB_1

	Inactive	I1	I2	I3	I4	Active
N-terminus motion (Å)	18.35 ± 0.49	17.12 ± 0.16	27.91 ± 1.21	20.12 ± 0.84	23.43 ± 0.18	30.72 ± 0.77
TM1 movement (Å)	23.72 ± 0.54	27.88 ± 0.37	24.28 ± 0.22	28.51 ± 0.25	26.96 ± 0.1	23.29 ± 0.39
TG relative motion (Å)	1.35 ± 0.13	-0.09 ± 0.47	1.9 ± 0.09	-3.8 ± 0.13	-4.57 ± 0.12	-3.66 ± 0.12
TM6 movement (Å)	11.01 ± 0.21	11.05 ± 0.2	10.78 ± 0.09	14.58 ± 0.16	15.26 ± 0.22	15.03 ± 0.21
TM7 movement (Å)	16.43 ± 0.48	15.81 ± 0.46	17.91 ± 0.24	9.37 ± 0.89	8.51 ± 0.24	9.77 ± 1.11

Supplementary Table 5: Mean and standard deviation of structurally important features for every metastable state of CB_2

	Inactive	I1	I2	I3	I4	Active
TM1 movement (Å)	26.05 ± 0.47	27.86 ± 0.41	24.06 ± 0.37	25.0 ± 0.33	21.85 ± 0.23	22.41 ± 0.49
TG rotation (Å)	71.97 ± 17.43	70.3 ± 9.15	76.04 ± 24.16	92.52 ± 9.99	91.79 ± 12.59	64.97 ± 11.29
TM5 movement (Å)	16.38 ± 0.79	15.37 ± 0.28	17.95 ± 0.29	18.55 ± 0.41	10.14 ± 0.36	9.7 ± 0.21
TM6 movement (Å)	10.42 ± 0.43	9.8 ± 0.19	9.83 ± 0.12	10.93 ± 0.33	15.22 ± 0.28	14.73 ± 0.24
TM7 movement (Å)	13.73 ± 0.41	14.85 ± 0.38	11.73 ± 0.42	12.92 ± 0.37	8.57 ± 0.14	8.82 ± 0.5

Supplementary Note 2. System preparation and analysis for long timescale apo MD simulation

System preparation and Simulation Details

Representative structures for each long timescale simulation were selected randomly from each metastable state. Only protein structures were preserved from the simulation, stripping out water (TIP3P), salt (Na+ and Cl-) and the membrane (POPC). Protein structures were again embedded in membrane (POPC) with salt solutions in extracellular and intracellular direction. Embedded structures were minimized and equilibrated before production run as described in the original set up. $\sim$ 600 ns of production run were performed on each structure.

RMSD and RMSF analysis

Structural analysis such as RMSD and RMSF were calculated using the AMBER tool CPPTRAJ v18.01.[16] Starting structure of the simulation were considered as the reference structure for RMSD and RMSF calculations. RMSD calculations were performed based on all backbone atoms of the residues.

tIC projection

Residue pair distances based on the RRCS were calculated for each frame of every simulation with python package MDTraj v1.9.3.[17] These distances were transformed into tIC projection using the weights of tIC object build on the adaptive sampling data for CB_1 and CB_2 separately.

Supplementary Note 3. System preparation and analysis for long timescale holo MD simulation

System Preparation and Simulation Details

Representing structures of ligand docked proteins were selected for MD simulations. Ligands were parameterized using GAFF forcefield with AmberTools antechamber.[18,19] The holo structures of the proteins were embedded in the POPC membrane with NaCl solution in the extracellular and intracellular portion. Protein and lipid were parameterized with AMBER ff14SB and lipid17 forcefield. TIP3P water model was used for water. Systems were minimized and equilibrated using our original workflow. $\sim$ 200 ns of production run was performed for each system.

RMSD and RMSF analysis

Structural analysis such as RMSD were calculated using the AmberTools CPPTRAJ v18.01.[16] Starting structure of the simulation were considered as the reference structure for RMSD calculations. All heavy atoms and hydrogens were selected for RMSD calculation.

Dynamic Contact Analysis

Python tool Getcontacts was used for calculation of the contacts between the ligand and receptors during MD simulation (`https://getcontacts.github.io/`). If a contact between the ligand and receptor is forming more than 50% of the MD frames, that contact is considered as a stable interaction.

Interaction Energy Calculation

The residues that are forming a stable contact with the ligands in either of the metastable states were considered for interaction energy calculation. Non-bonded energy calculations

between the protein and the ligand were perfumed using Linear interaction energy (LIE) method implemented in AmberTools.[20] In total energy contribution, both van der waals (vdw) and electrostatic energy contributions were considered.

Supplementary Table 6: Mean and standard error of the calculated ligand RMSD distribution for CB_1 (Intermediate state 2 and Active state) and CB_2 (Inactive and active state)

Ligands	CB_1 (I2 state) (Å)	CB_1 (Active state) (Å)	CB_2 (Inactive state) (Å)	CB_2 (Active state) (Å)
JWH-133	1.70 ± 0.00	0.72 ± 0.00	0.61 ± 0.00	0.62 ± 0.00
HU-308	1.73 ± 0.00	1.33 ± 0.00	0.87 ± 0.00	1.03 ± 0.00
JWH-015	1.77 ± 0.00	0.94 ± 0.28	1.02 ± 0.01	1.41 ± 0.00
AM1241	1.36 ± 0.00	1.49 ± 0.00	0.89 ± 0.00	2.03 ± 0.00

Supplementary Table 7: Contact frequencies and corresponding interaction energies (vdw + electrostatic) of the residues that form stable contacts with the ligand for both macrostates (inactive and active) of CB_2. Interaction energy calculations were performed with LIE method in Ambertools.

Residues	Inactive	Active
	JWH-133	
$C288^{7.42}$	0.642 (-1.33)	0.032 (-0.21)
	HU-308	
$F106^{3.25}$	0.64(-1.36)	0.00(-0.70)
$V113^{3.32}$	0.90(-3.42)	0.12(-1.69)
$L182^{ECL2}$	0.09(-1.69)	0.82(-4.62)
	JWH-015	
$F91^{2.61}$	0.22(-3.17)	0.86(-4.34)
	AM1241	
$H95^{2.65}$	1.0(-7.70)	0.01(-0.63)
$V113^{3.32}$	0.98(-1.90)	0.37(-2.74)
$K278^{7.32}$	0.0(-0.06)	0.72(0.75)

Supplementary Table 8: Distance features used to build Markov state model for CB_1 using RRCS.

Position	Number	Type	Feature
Extracellular	1	$C\alpha$ distance	$E100^{N-term}$-$H270^{ECL2}$
	2		$N101^{N-term}$-$F177^{2.64}$
	3		$N101^{N-term}$-$K183^{ECL1}$
	4		$N101^{N-term}$-$D184^{ECL1}$
	5		$F102^{N-term}$-$F189^{3.25}$
	6		$F102^{N-term}$-$K192^{3.28}$
	7		$M103^{N-term}$-$F170^{2.57}$
	8		$M103^{N-term}$-$S173^{2.60}$
	9		$M103^{N-term}$-$F174^{2.61}$
	10		$D104^{N-term}$-$F177^{2.64}$
	11		$F108^{N-term}$-$D266^{ECL2}$
	12		$F108^{N-term}$-$F268^{ECL2}$
	13		$F108^{N-term}$-$P269^{ECL2}$
	14		$M109^{N-term}$-$F177^{2.64}$
	15		$M109^{N-term}$-$H181^{ECL1}$
	16		$M109^{N-term}$-$Q115^{1.31}$
	17		$M109^{N-term}$-$Q116^{1.32}$
	18		$V110^{N-term}$-$H178^{2.65}$
	19		$A120^{1.36}$-$F174^{2.61}$
	20		$L122^{1.38}$-$M384^{7.40}$
	21		$R186^{3.22}$-$N256^{ECL2}$
	22		$R186^{3.22}$-$E258^{ECL2}$
	23		$F191^{3.27}$-$L252^{4.61}$
	24		$P251^{4.60}$-$Y275^{5.39}$

	25		G254$^{4.63}$-K259^{ECL2}
	26		E258^{ECL2}-H270^{ECL2}
	27		S262^{ECL2}-E273$^{5.37}$
	28		D266^{ECL2}-M371^{ECL3}
Transmembrane	29		V131$^{1.47}$-S167$^{2.54}$
	30		E133$^{1.49}$-P394$^{7.50}$
	31		F200$^{3.36}$-W356$^{6.48}$
	32		F200$^{3.36}$-C386$^{7.42}$
	33		T201$^{3.37}$-A244$^{4.53}$
	34		L207$^{3.43}$-Y294$^{5.58}$
	35		F208$^{3.44}$-L286$^{5.50}$
	36		L209$^{3.45}$-F237$^{4.46}$
	37		C355$^{6.47}$-L385$^{7.41}$
	38		W356$^{6.48}$-C386$^{7.42}$
Intracellular	39		S144$^{1.60}$-Y153$^{2.40}$
	40		L147^{ICL1}-Y153$^{2.40}$
	41		R148^{ICL1}-D403$^{8.49}$
	42		P151$^{2.38}$-D403$^{8.49}$
	43		H154$^{2.41}$-F237$^{4.46}$
	44		F155$^{2.42}$-T210$^{3.46}$
	45		F155$^{2.42}$-Y397$^{7.53}$
	46		F155$^{2.42}$-F237$^{4.46}$
	47		R214$^{3.50}$-Y294$^{5.58}$
	48		R214$^{3.50}$-D338$^{6.30}$
	49		A301$^{5.65}$-R340$^{6.33}$

	50		H304$^{5.68}$-A335$^{6.27}$
	51		H304$^{5.68}$-D338$^{6.30}$
	52		A335$^{6.27}$-R340$^{6.32}$
	53		K343$^{6.35}$-R400$^{7.56}$
	54		T344$^{6.36}$-R400$^{7.56}$

Supplementary Table 9: Distance features used to build Markov state model for CB_2 using RRCS.

Position	Number	Type	Feature
Extracellular	1	Cα distance	M22^{N-term}-F106$^{3.25}$
	2		I27^{N-term}-D275$^{7.29}$
	3		L28^{N-term}-H98^{ECL1}
	4		K33$^{1.32}$-V96$^{2.66}$
	5		W172^{ECL2}-Y190$^{5.39}$
	6		W172^{ECL2}-R177^{ECL2}
	7		W172^{ECL2}-D189$^{5.38}$
	8		T173^{ECL2}-L285^{ECL2}
	9		C174^{ECL2}-L285^{ECL2}
	10		R177^{ECL2}-P187$^{5.36}$
	11		R177^{ECL2}-N188$^{5.37}$
	12		R177^{ECL2}-D189$^{5.38}$
	13		E181^{ECL2}-K278$^{7.32}$
	14		N188$^{5.37}$-L269$^{6.59}$
	15		D101^{ECL1}-K109$^{3.28}$
	16		L107$^{3.26}$-L169$^{4.61}$
	17		L264$^{6.54}$-F281$^{7.35}$
Transmembrane	18		N51$^{1.50}$-F81$^{2.51}$
	19		D80$^{2.50}$-S292$^{7.46}$
	20		A83$^{2.53}$-F117$^{3.36}$
	21		F117$^{3.36}$-C288$^{7.42}$
	22		L124$^{3.43}$-Y299$^{7.53}$
	23		Y209$^{5.58}$-G248$^{6.38}$
	24		L254$^{6.44}$-N291$^{7.45}$

Intracellular	25		L64^{ICL1}-Y70$^{2.40}$
	26		R66^{ICL1}-R147$^{4.39}$
	27		Y70$^{2.40}$-E305$^{8.49}$
	28		F72$^{2.42}$-L126$^{3.45}$
	29		F72$^{2.42}$-T127$^{3.46}$
	30		A128$^{3.47}$-Y209$^{5.58}$
	31		R131$^{3.50}$-Y209$^{5.58}$
	32		R131$^{3.50}$-Y299$^{7.53}$
	33		R131$^{3.50}$-L243$^{6.33}$
	34		L133$^{3.52}$-Y141^{ICL2}
	35		L135$^{3.54}$-R242$^{6.32}$
	36		K245$^{6.35}$-R302$^{7.56}$
	37		T246$^{6.36}$-R302$^{7.56}$
	38		A300$^{7.54}$-R307$^{8.51}$

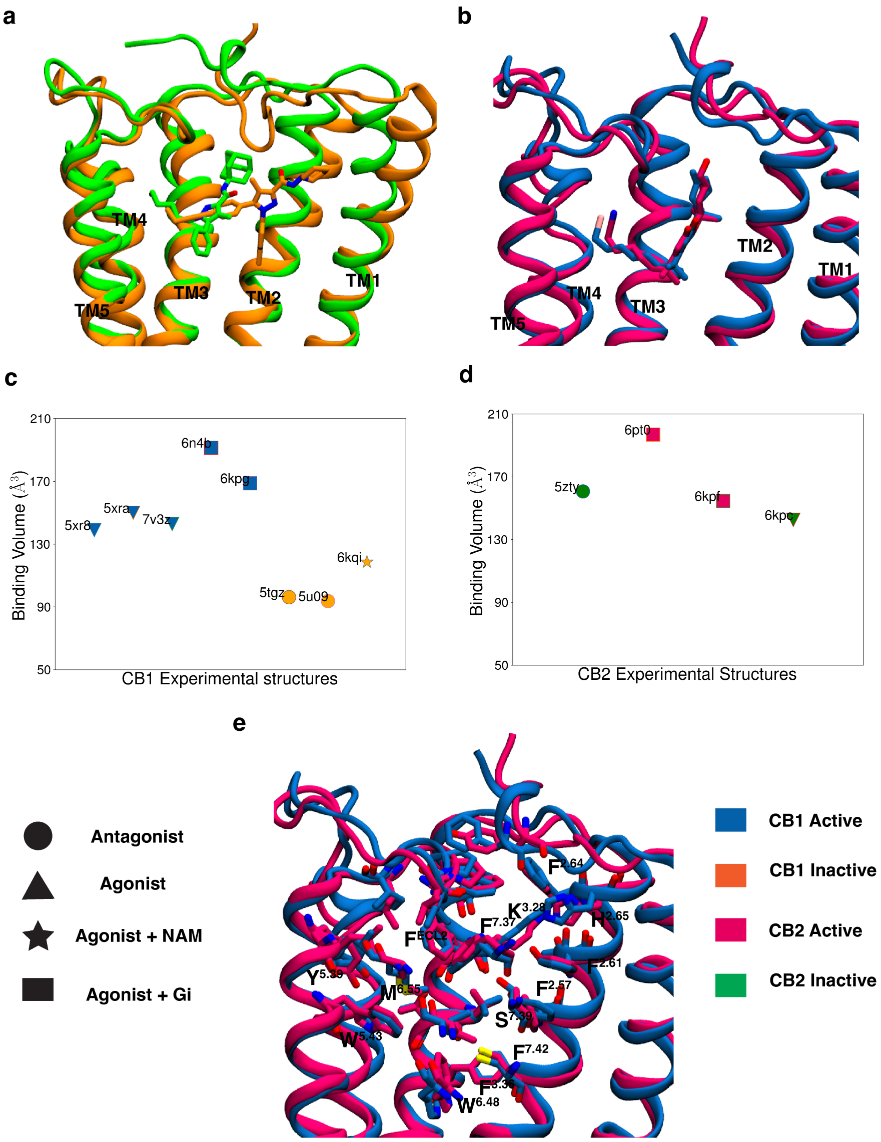

Supplementary Figure 1: Superposition of inactive (**a**) and active (**b**) structures of CB_1 (Active PDB ID: 5XRA, color: Orange; Inactive PDB ID: 5TGZ, color: Green) and CB_2 (Active PDB ID: 6KPF, color: Purple; Inactive PDB ID: 5ZTY, color: Blue). Proteins are shown as cartoon representation. Agonists (CB_1 agonist: AM11542; CB_2 agonist: AM841) antagonists (CB_1 antagonist: AM6538; CB_2 antagonist: AM10257) are shown as sticks. Cartoon representations of TM5 and TM6 are not shown for better visualization of ligand bound positions. Binding pocket volumes for experimentally determined CB_1 (**c**) and CB_2 (**d**) structures are shown as scatter plots. Markers are colored based on the activation state of the protein (CB_1 active: blue, CB_1 inactive: orange, CB_2 active: purple, CB_2 inactive: green). The shape of the marker is based on the type of the ligand and downstream signaling partner (Antagonist bound: circle, Agonist bound: triangle, Agonist and NAM bound: star, Agonist and G protein bound: rectangle). **e** Superposition of active CB_1 (PDB ID: 5XRA, color: blue) and CB_2 (PDB ID: 6KPF, color: purple) structures. Protein structures are shown as cartoon representation. Binding pocket residues are shown as sticks. Cartoon representation of TM6 and TM7 are not shown for better visualization.

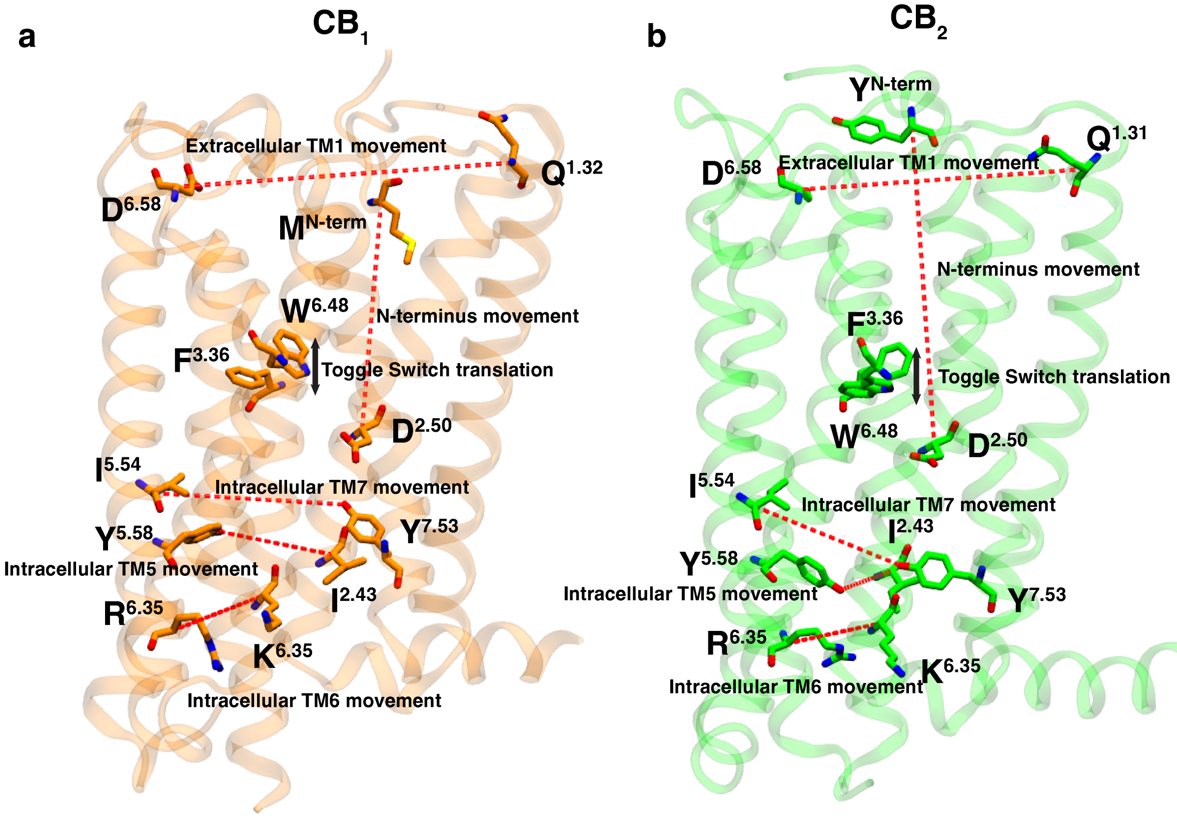

Supplementary Figure 2: Inactive crystal structure of CB_1 (**a**) (PDB ID: 5TGZ, color: Orange) and CB_2 (**b**) (PDB ID: 5TZY, color: Green) are shown as cartoon representations. Distance metrics used to calculate conserved and non-conserved structural differences between active and inactive states are shown red dotted lines. Toggle switch movements captured by z component difference between $W^{6.48}$ and $F^{3.36}$ are represented as bidirectional arrow.

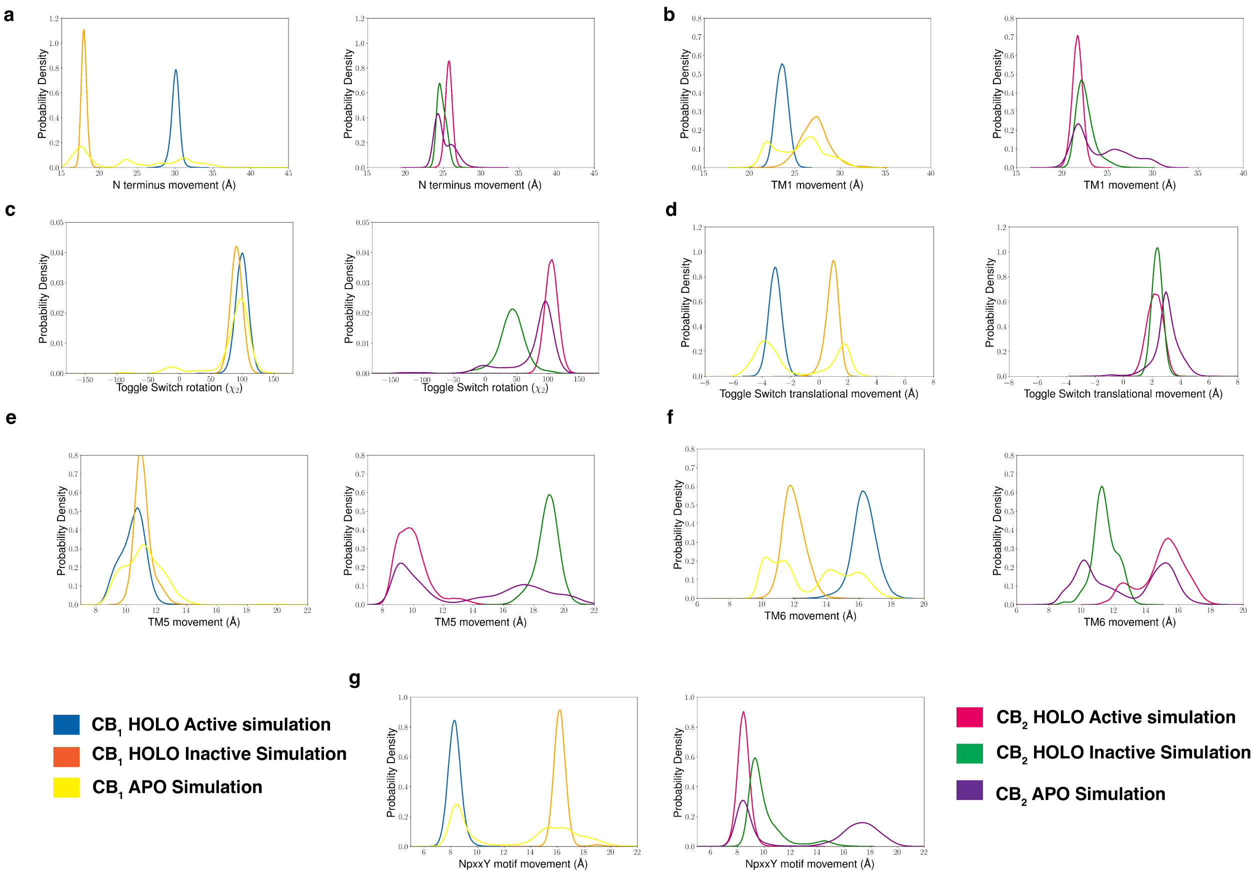

Supplementary Figure 3: Comparison of distribution of non-conserved (**a**, **b**, **c**, **d**, **e**) and conserved (**f**, **g**) changes of CB_1 and CB_2 structural features obtained from apo (without ligand) and holo (agonist and antagonist bound) simulation.

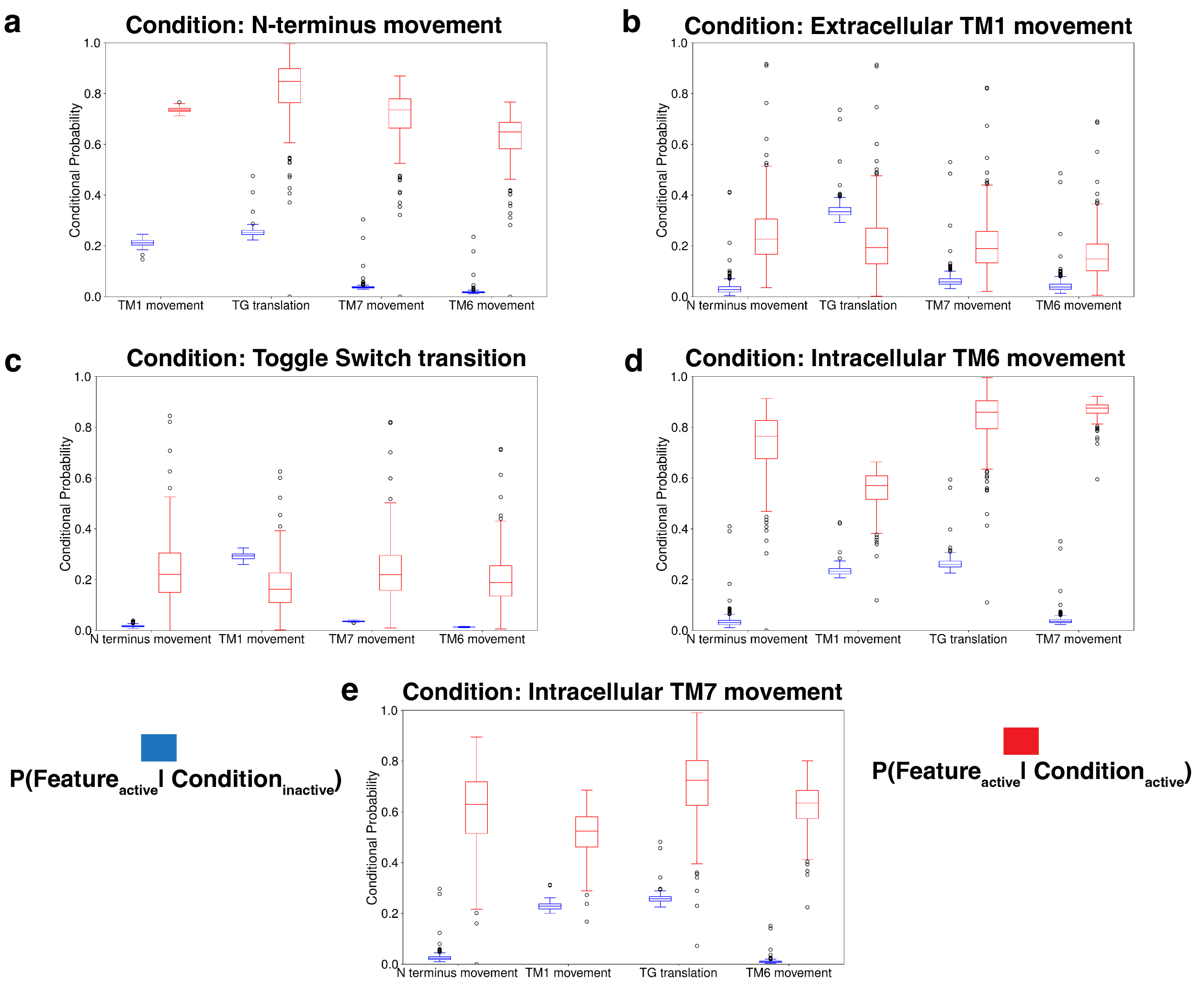

Supplementary Figure 4: Box plot to show the conditional probabilities for all combinations of structurally important features for CB_1. Blue box represents the condition probability for active features when the conditional features are inactive. Red box represents the condition probability for active features when the conditional features are active. Error calculations were performed based on bootstrapping. 200 bootstrap samples were selected with 80% of the total number of trajectories.

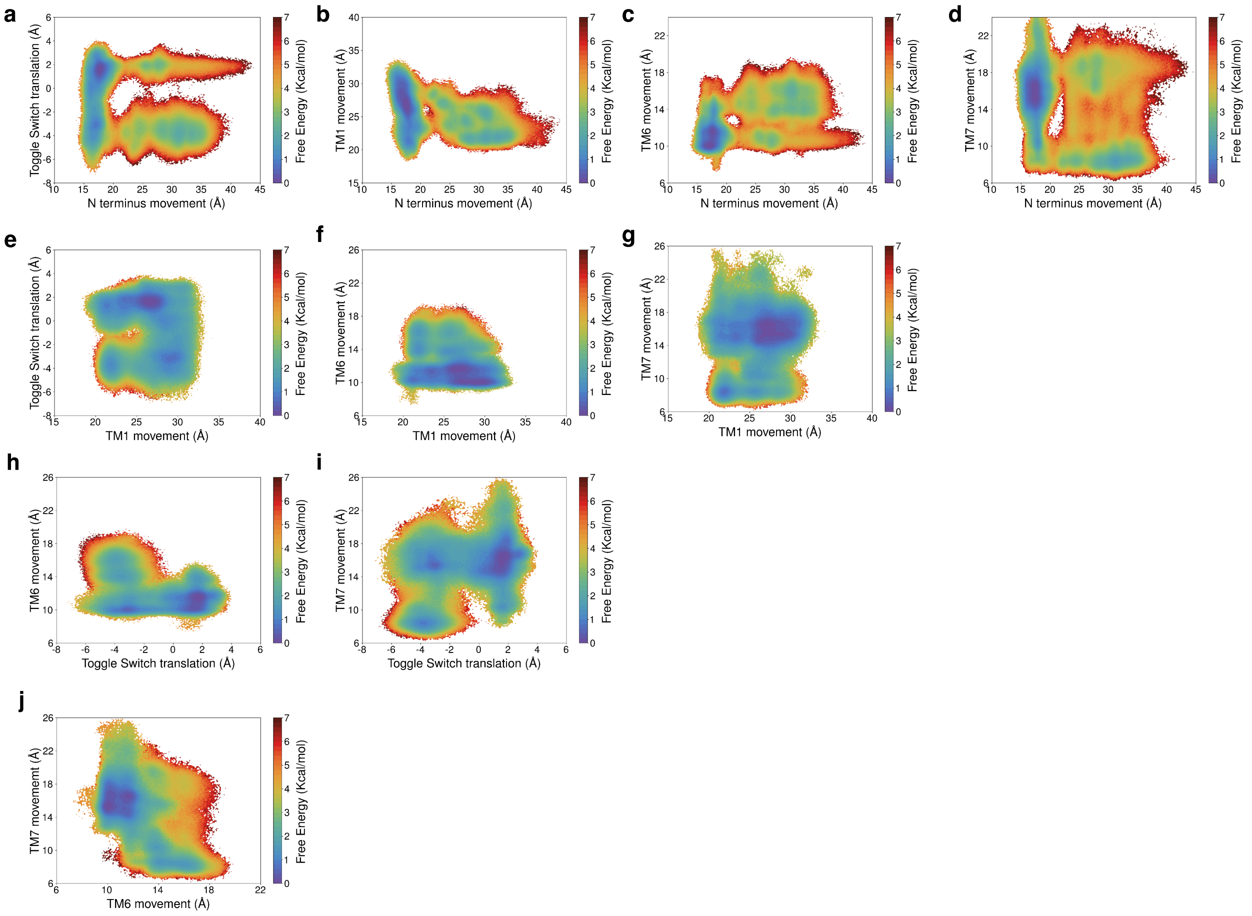

Supplementary Figure 5: MSM weighted free energy landscapes for all the combinations of dynamically important features for CB_1 as discussed in manuscript. **a**, **b**, **c**, **d** show the landscapes with x-axis representing N-terminus movement and y-axis representing other important movements. **e**, **f**, **g** show the landscapes with x-axis representing extracellular TM1 movement and y-axis representing other important movements. **h**, **i** show the landscapes with x-axis representing toggle switch translational movement and y-axis representing other important movements. **j** shows the landscape with x-axis representing intracellular TM6 movement and y-axis representing intracellular TM7 movement.

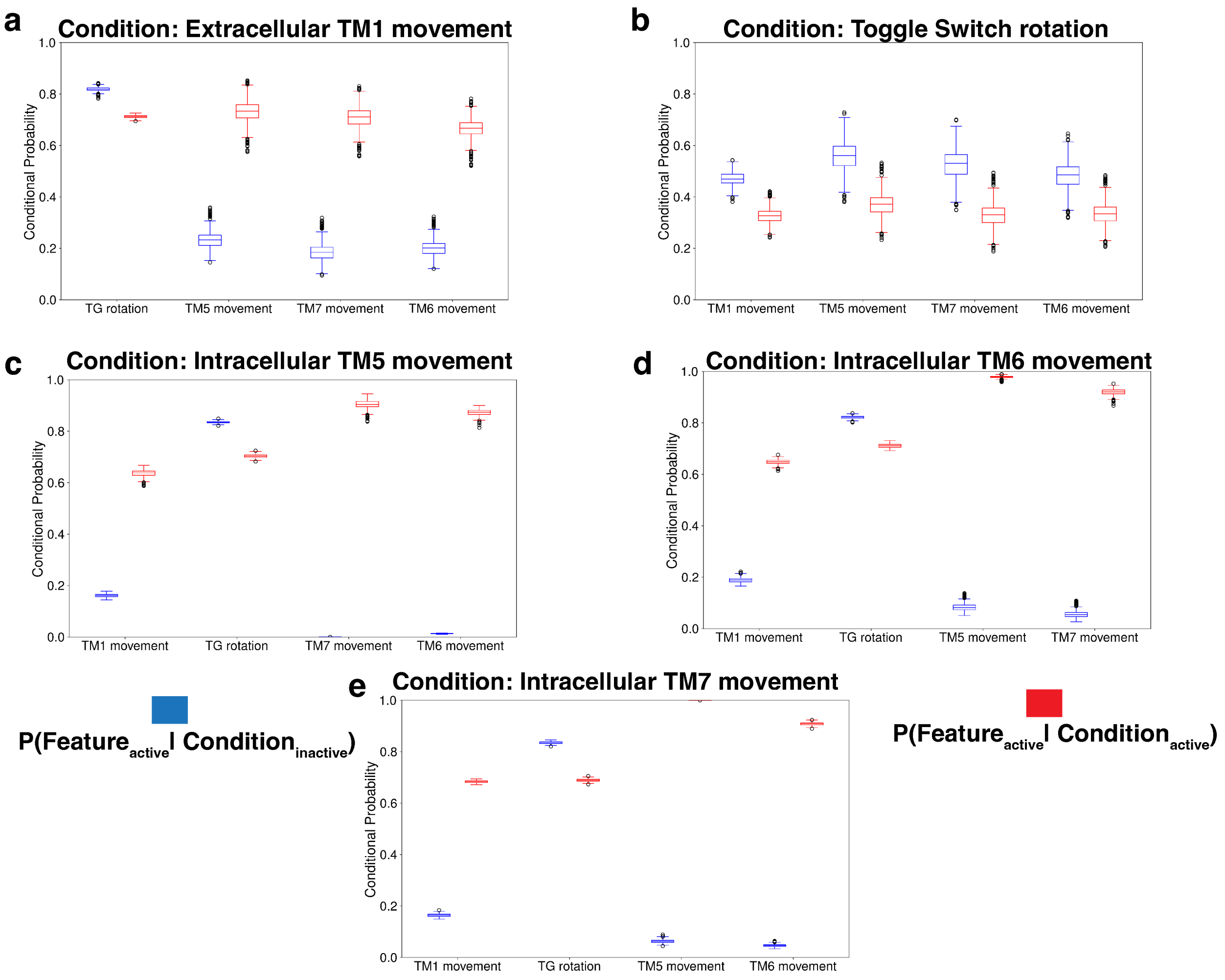

Supplementary Figure 6: Box plot to show the conditional probabilities for all combinations of structurally important features for CB_2. Blue box represents the condition probability for active features when the conditional features are inactive. Red box represents the condition probability for active features when the conditional features are active. Error calculations were performed based on bootstrapping. 200 bootstrap samples were selected with 80% of the total number of trajectories.

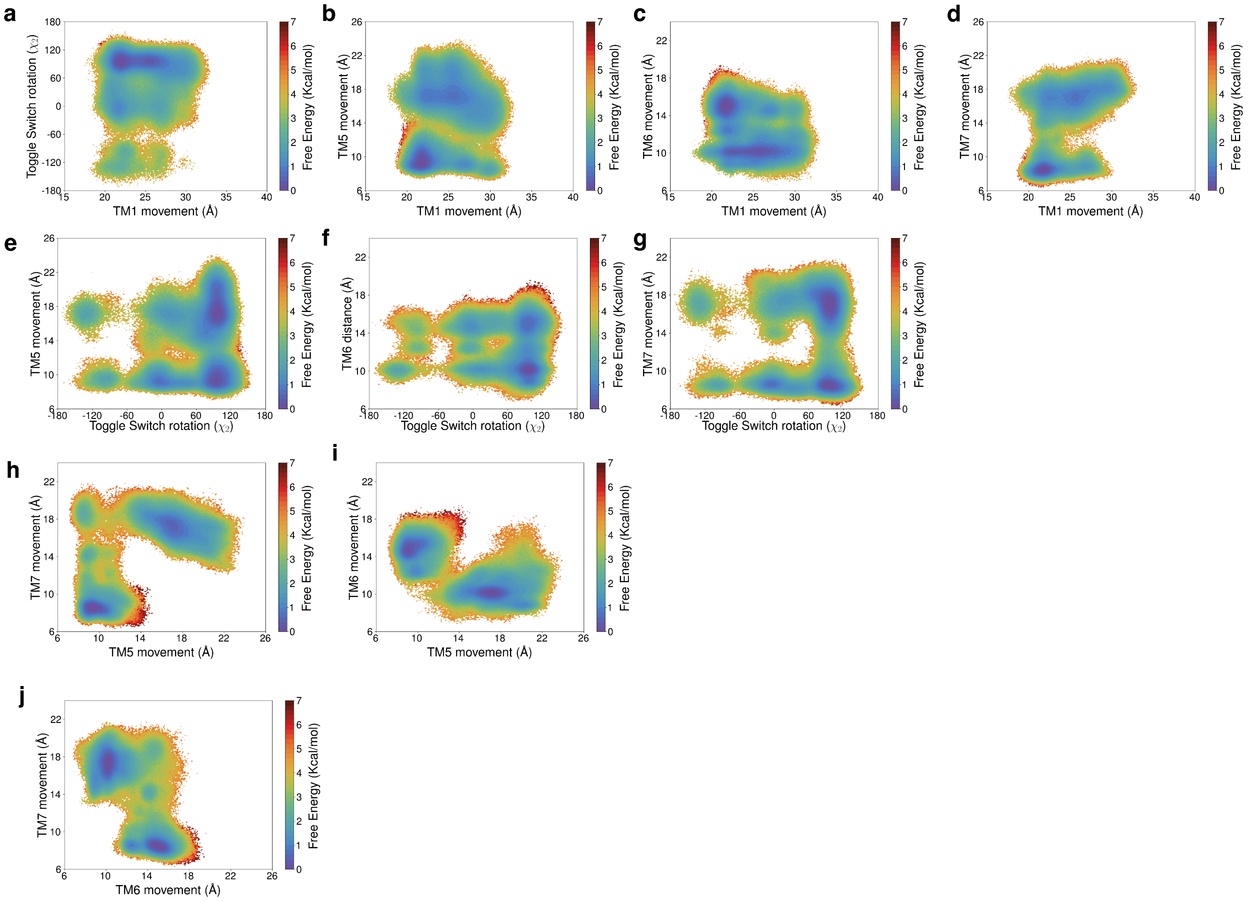

Supplementary Figure 7: MSM weighted free energy landscapes for all the combinations of dynamically important features as discussed for CB_2 in manuscript. **a**, **b**, **c**, **d** show the landscapes with x-axis representing extracellular TM1 movement and y-axis representing other important movements. **e**, **f**, **g** show the landscapes with x-axis representing extracellular toggle switch rotational movement and y-axis representing other important movements. **h**, **i** show the landscapes with x-axis representing intracellular TM5 movement and y-axis representing other important movements. **j** shows the landscape with x-axis representing intracellular TM6 movement and y-axis representing intracellular TM7 movement.

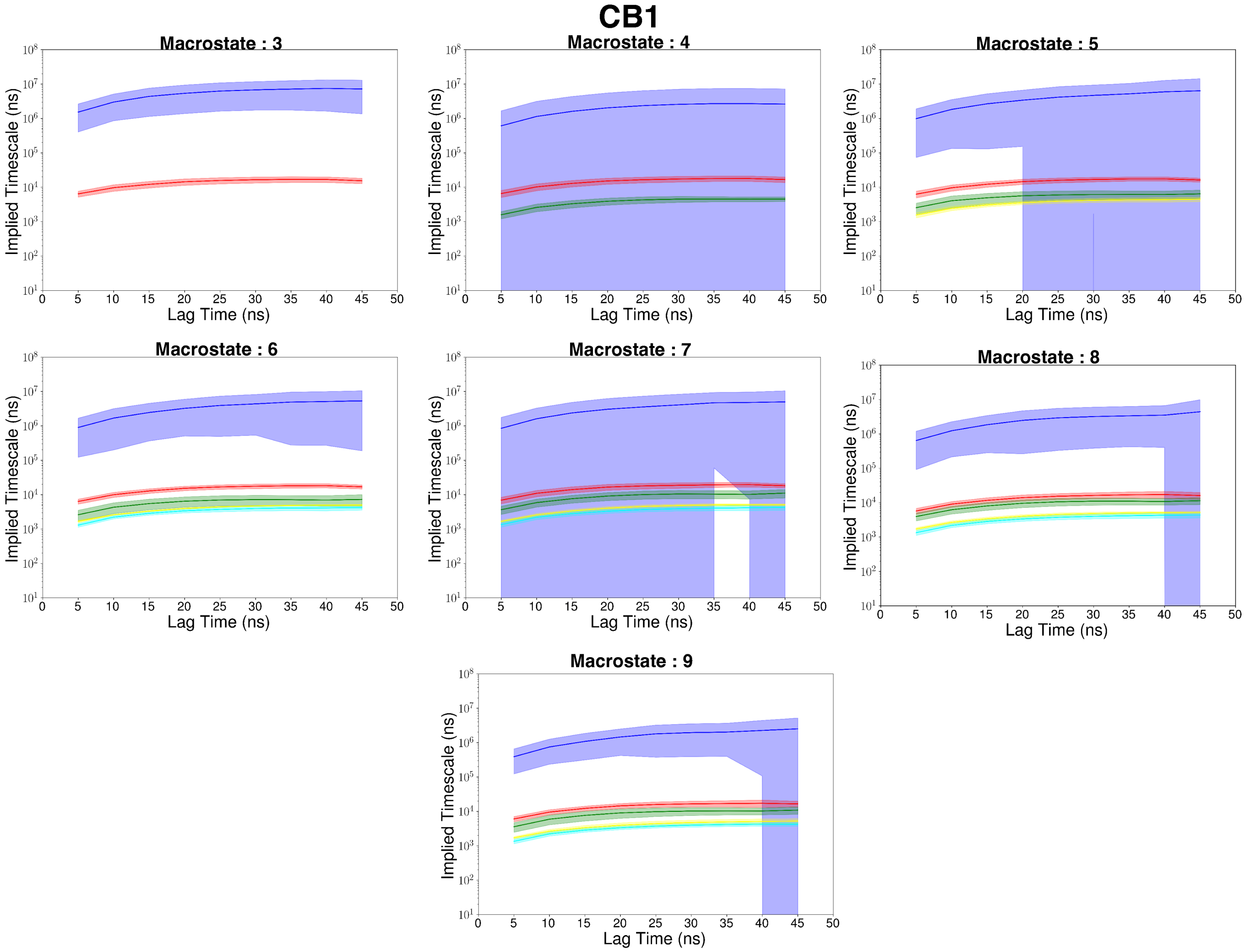

Supplementary Figure 8: For CB_1, implied timecale of VAMPnet models are plotted against the lag time based on the number of the macrostates selected to build the model. Error bars are calculated based on bootstrapping. 20 bootstrap samples were selected with 80% of the total number of trajectories.

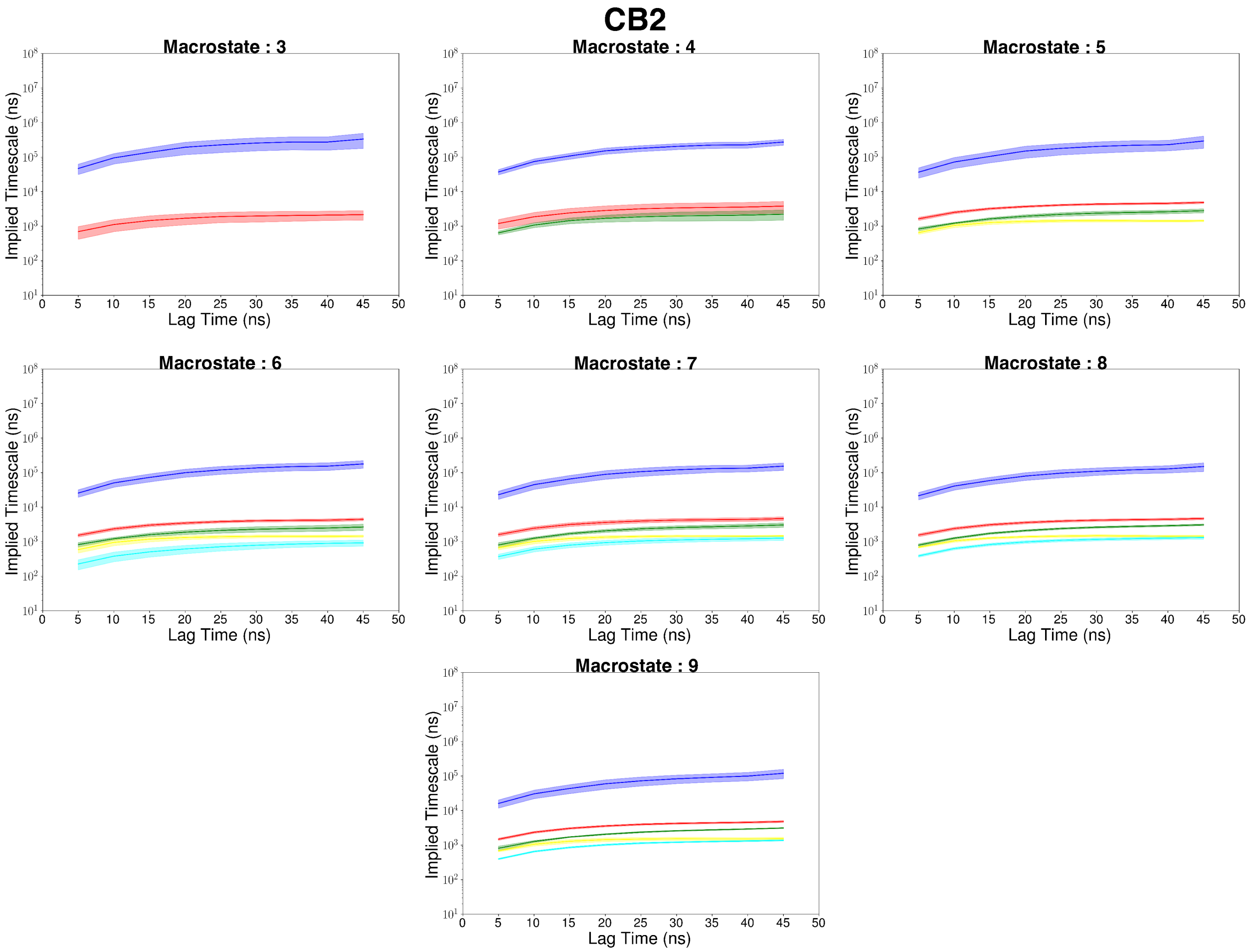

Supplementary Figure 9: For CB_2, implied timecale of VAMPnet models are plotted against the lag time based on the number of the macrostates selected to build the model. Error calculations were performed based on bootstrapping. 20 bootstrap samples were selected with 80% of the total number of trajectories.

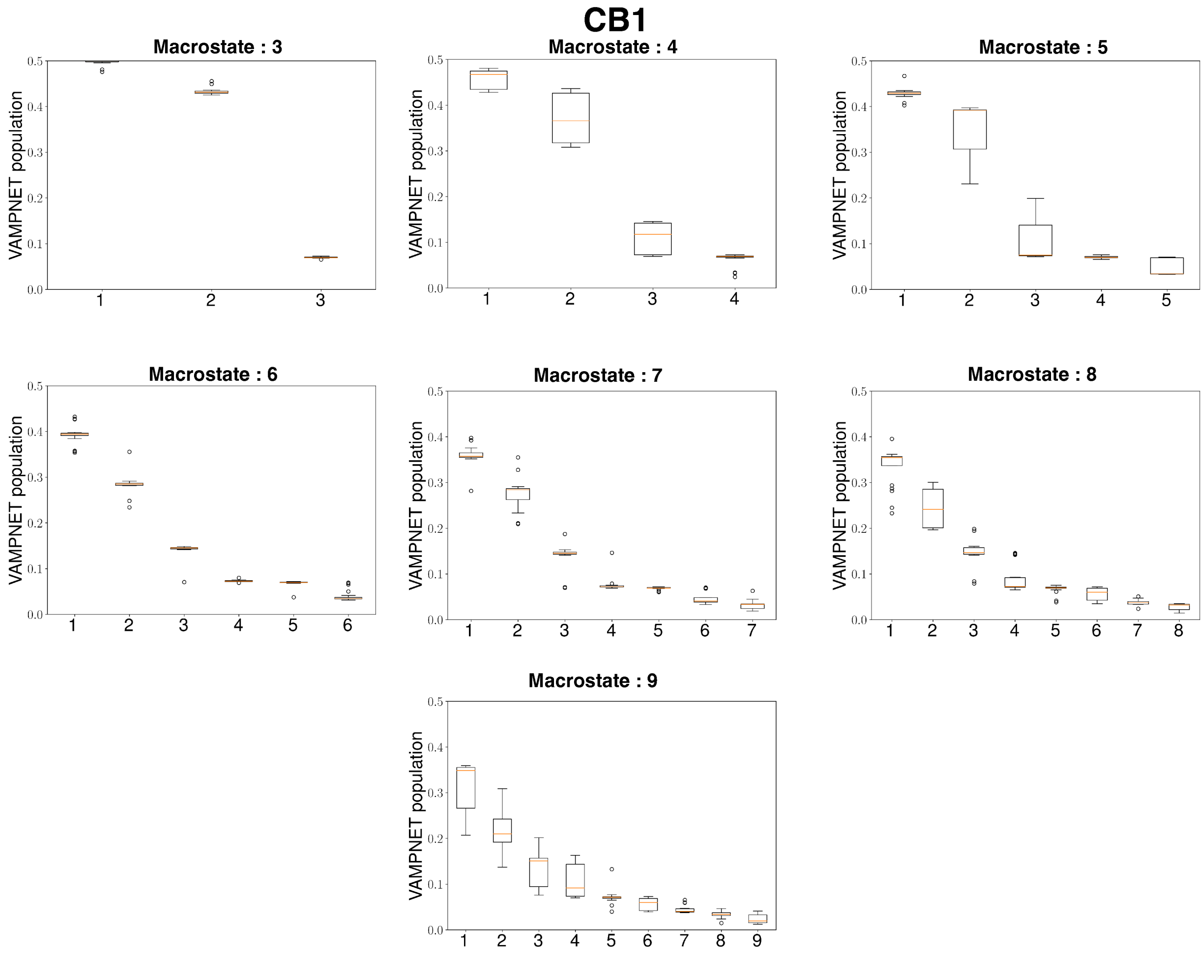

Supplementary Figure 10: For CB_1, state population of VAMPnet models are shown as box plots based on the number of the macrostates selected to build the model. Error calculations were performed based on bootstrapping. 20 bootstrap samples were selected with 80% of the total number of trajectories.

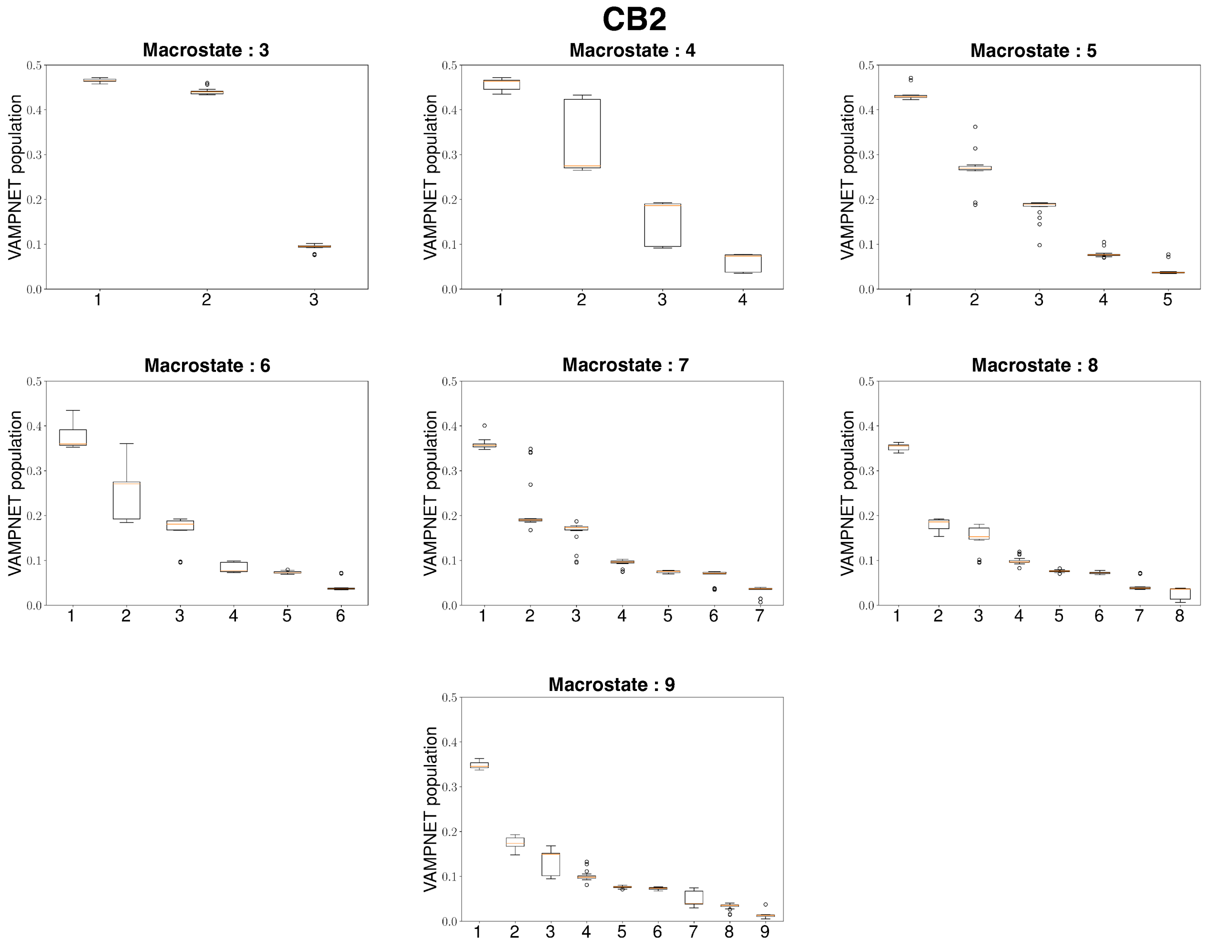

Supplementary Figure 11: For CB_2, state population of VAMPnet models are shown as box plots based on the number of the macrostates selected to build the model. Error bars are calculated based on bootstrapping. 20 bootstrap samples were selected with 80% of the total number of trajectories.

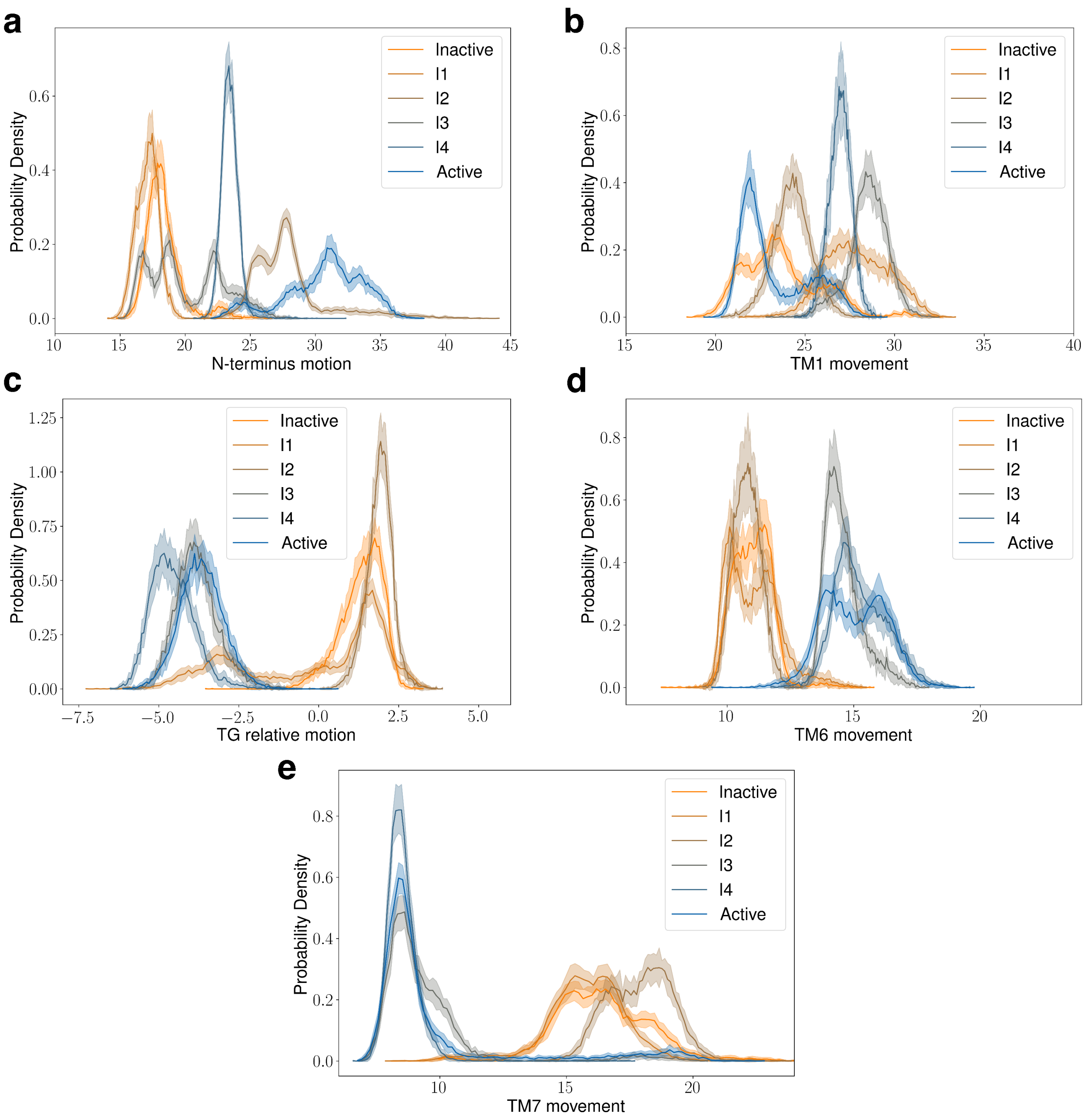

Supplementary Figure 12: Distributions of structurally important features (N-terminus movement (**a**), Extracellular TM1 movement (**b**), toggle switch translational movement (**c**), intracellular TM6 movement (**d**), intracellular TM7 movement (**e**)) for each metastable state of CB_1. Colors for metastable states change from orange to blue gradually from inactive to active state.

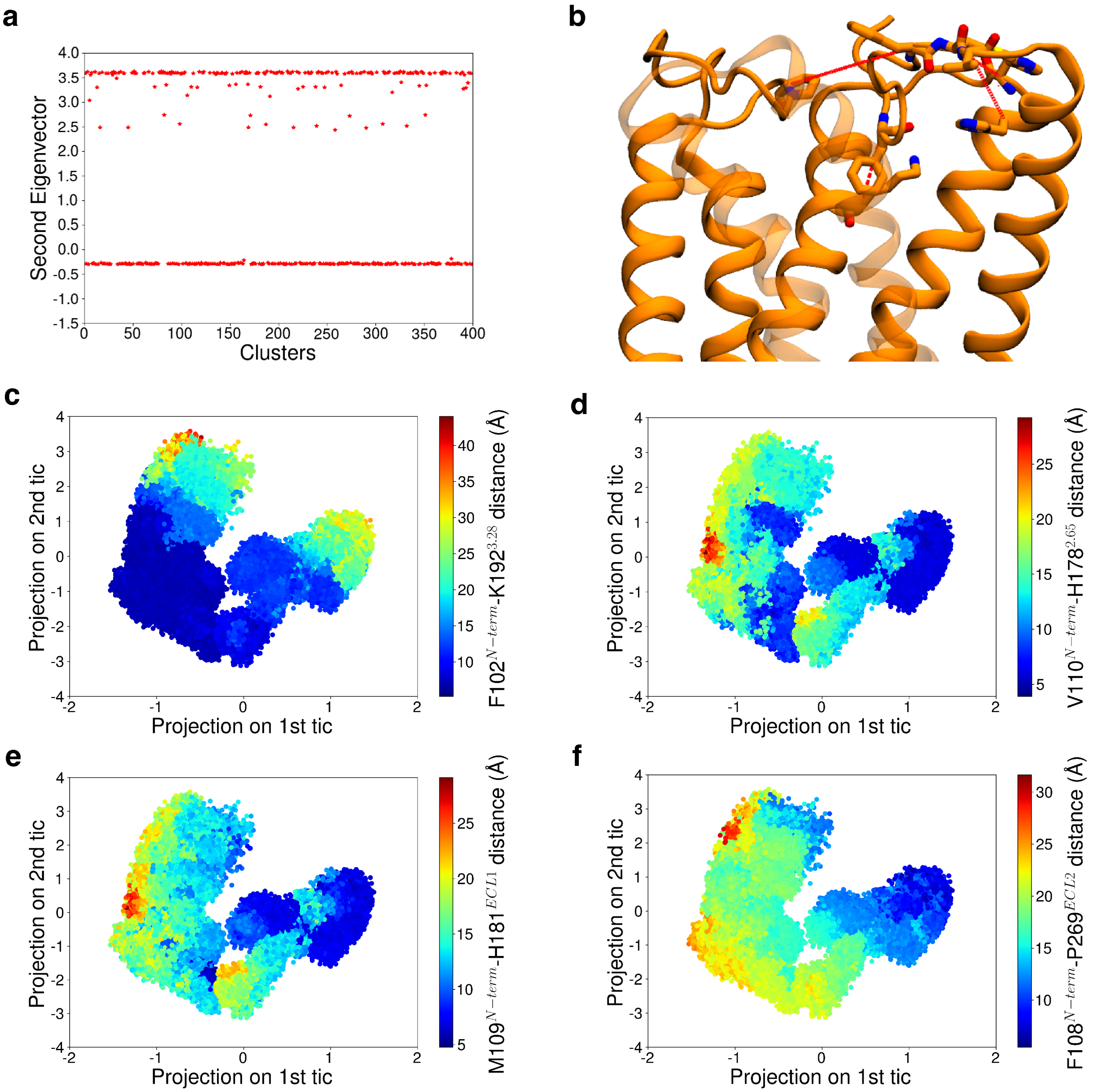

Supplementary Figure 13: (**a**) Values of the second eigenvectors of CB_1 MSM are plotted as a scatter plot against the cluster numbers. (**b**) Features that are correlated with the second eigenvectors are calculated are shown as red dotted line. (**c**, **d**, **e**, **f**) Scatter plots of tIC 1 and tIC 2 projections of CB_1 which are colored based on the values of four features that are highly correlated with second eigenvectors.

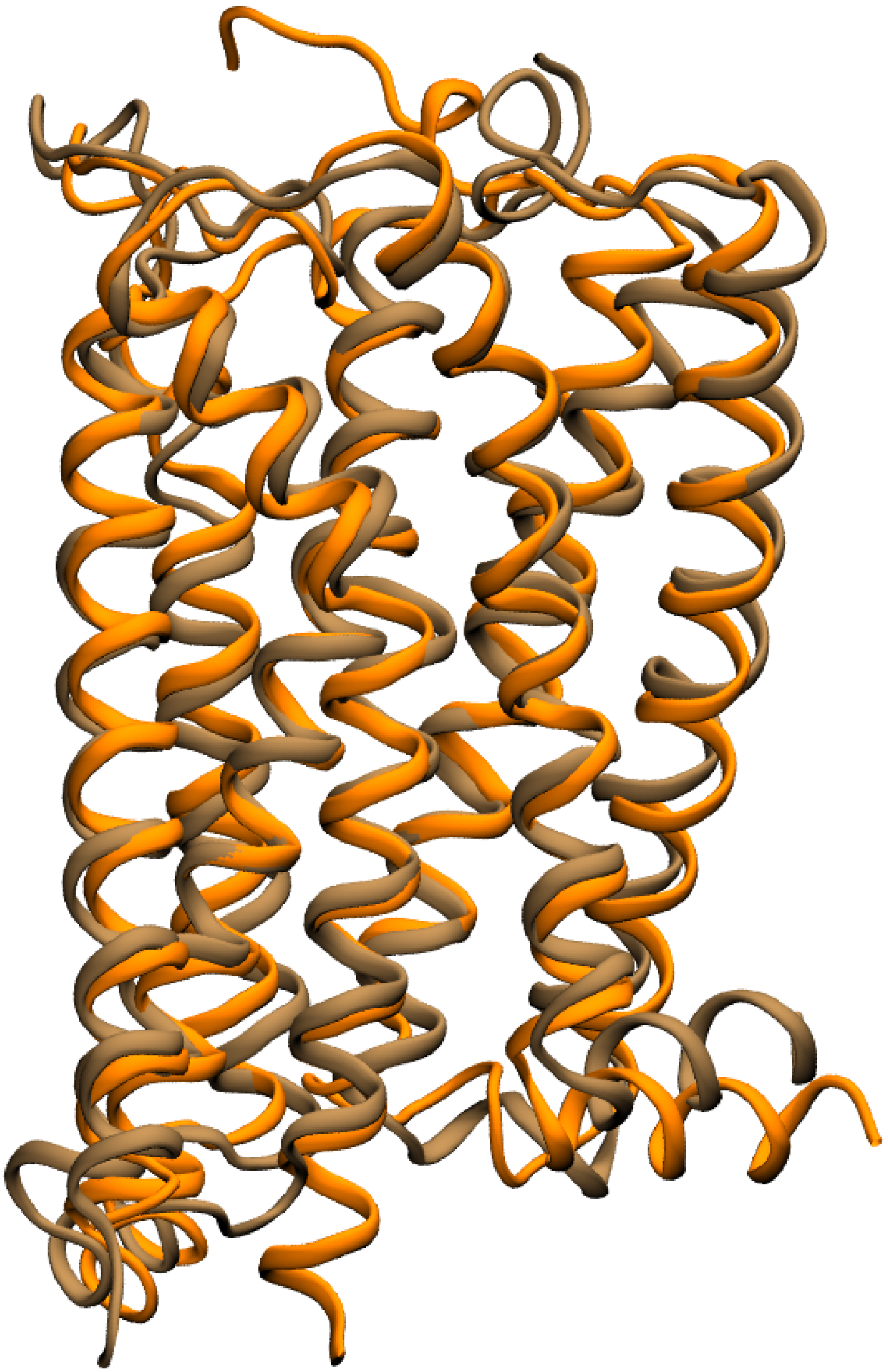

Supplementary Figure 14: Structural comparison of agonist and NAM bound PDB structure (6KQI, color: Orange) and representative structure from CB_1 I2 metastable state.

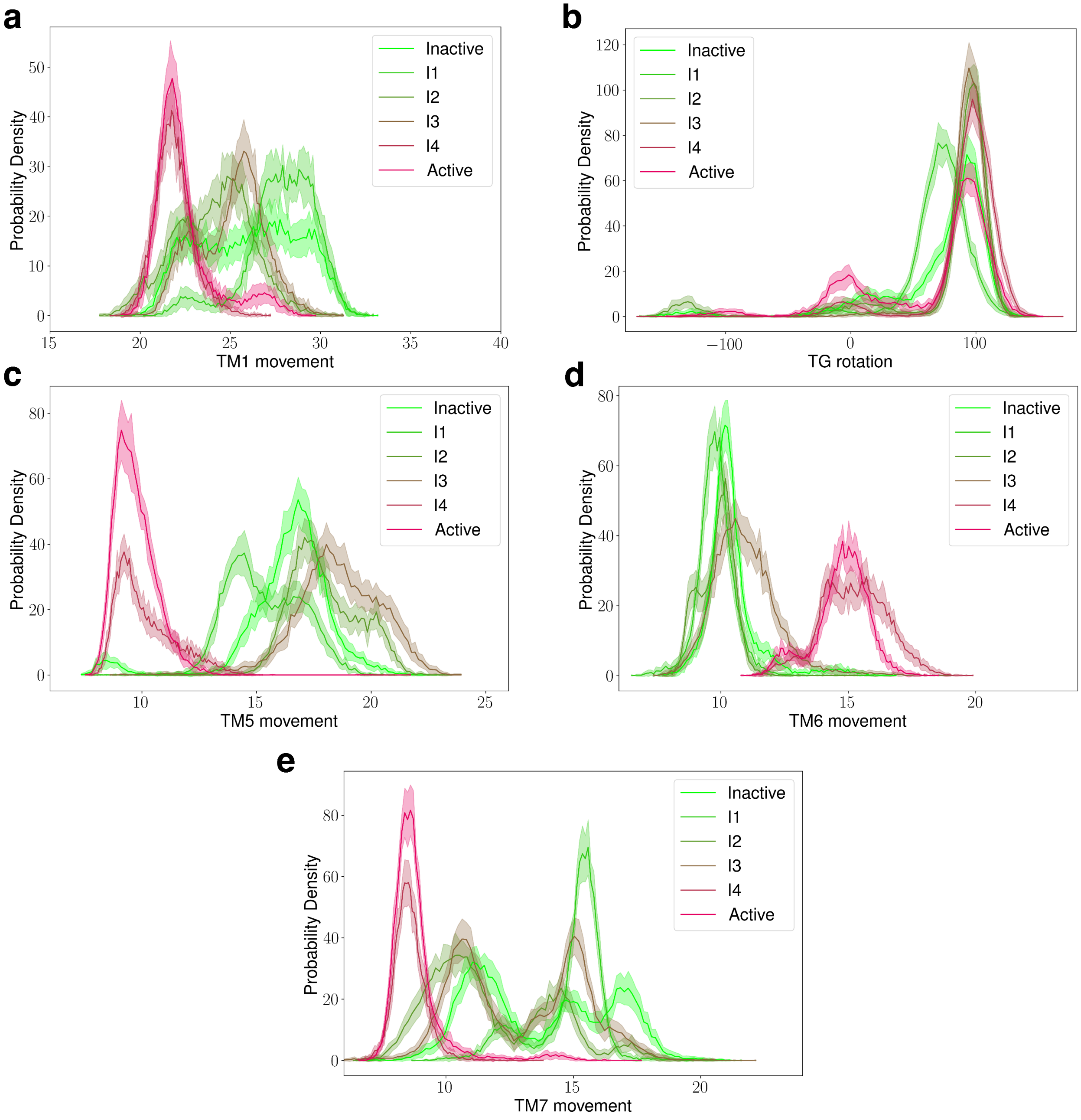

Supplementary Figure 15: Distributions of structurally important features (Extracellular TM1 movement (**a**), toggle switch rotational movement (**b**), intracellular TM6 movement (**c**), intracellular TM6 movement (**d**), intracellular TM7 movement (**e**)) for each metastable state of CB_2. Colors for metastable states change from green to purple gradually from inactive to active state.

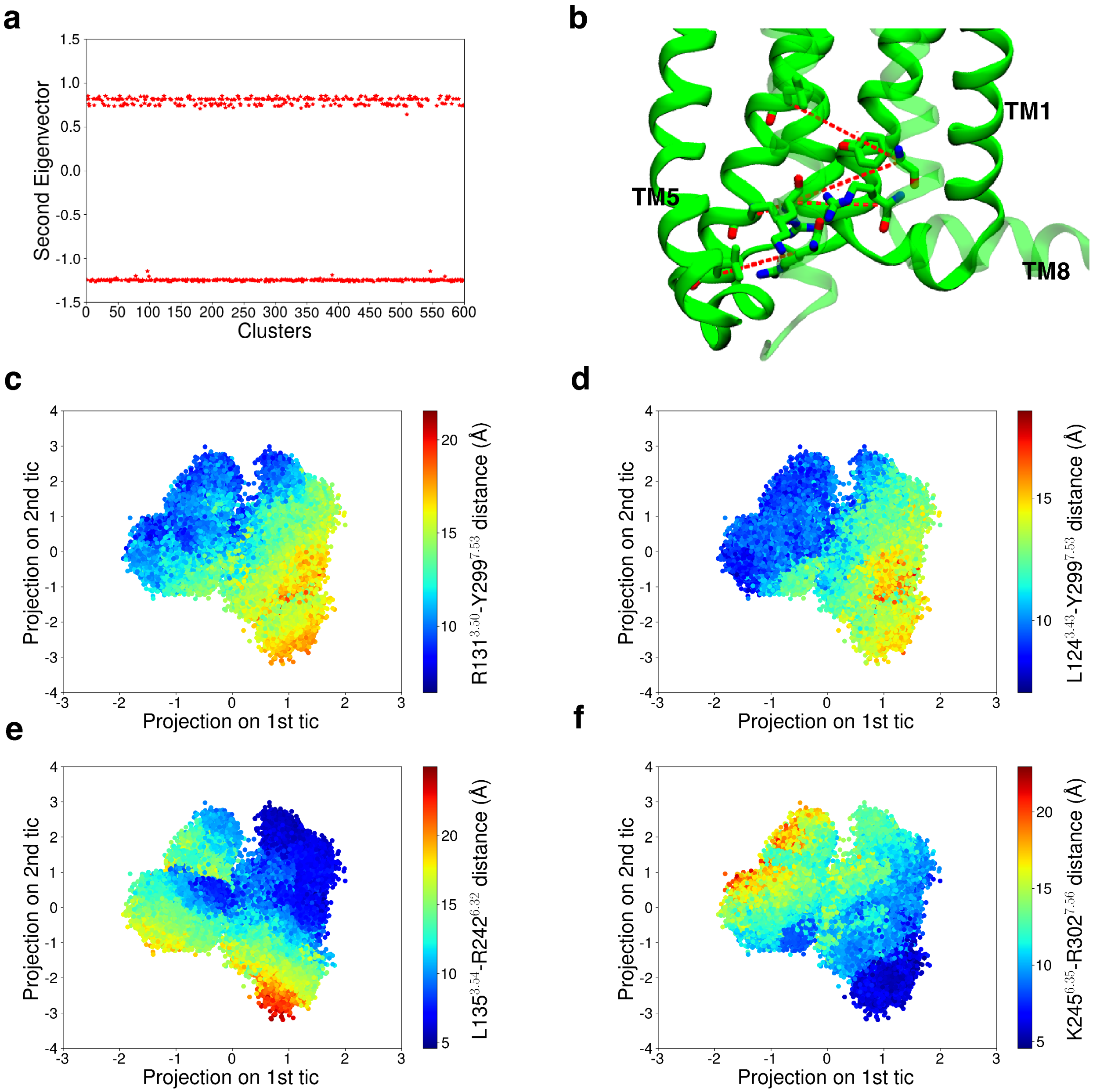

Supplementary Figure 16: (**a**) Values of the second eigenvectors of CB_2 MSM are plotted as a scatter plot against the cluster numbers. (**b**) Features that are correlated with the second eigenvectors are calculated are shown as red dotted line. (**c**, **d**, **e**, **f**) Scatter plots of tIC 1 and tIC 2 projections of CB_2 which are colored based on the values of four features that are highly correlated with second eigenvectors.

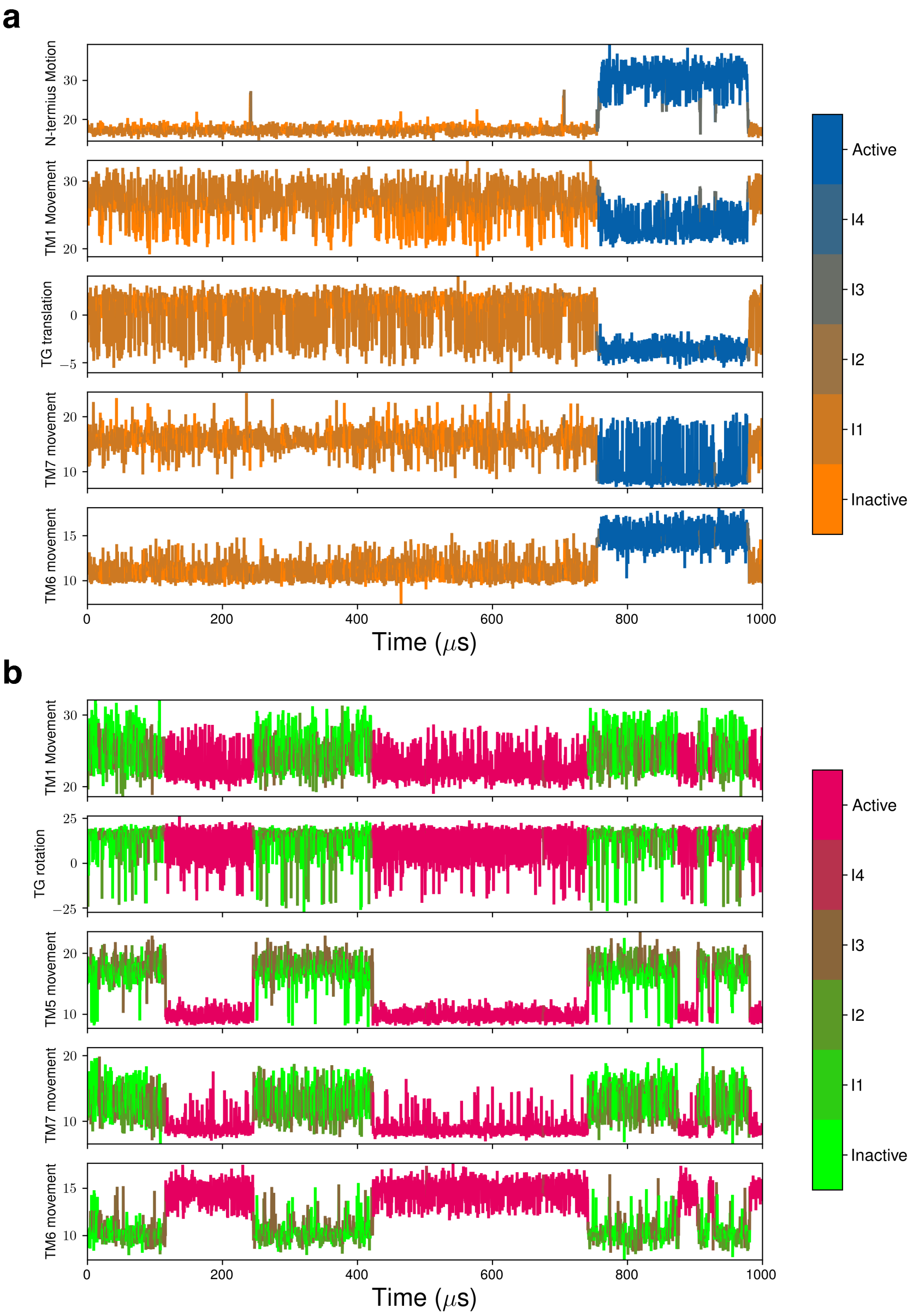

Supplementary Figure 17: Representative Kinetic monte carlo (kMC) simulation for CB_1 (**a**) and CB_2 (**b**) starting from the inactive state showing the transition of important structural features between different metastable states. Color bar represents different metastable states.

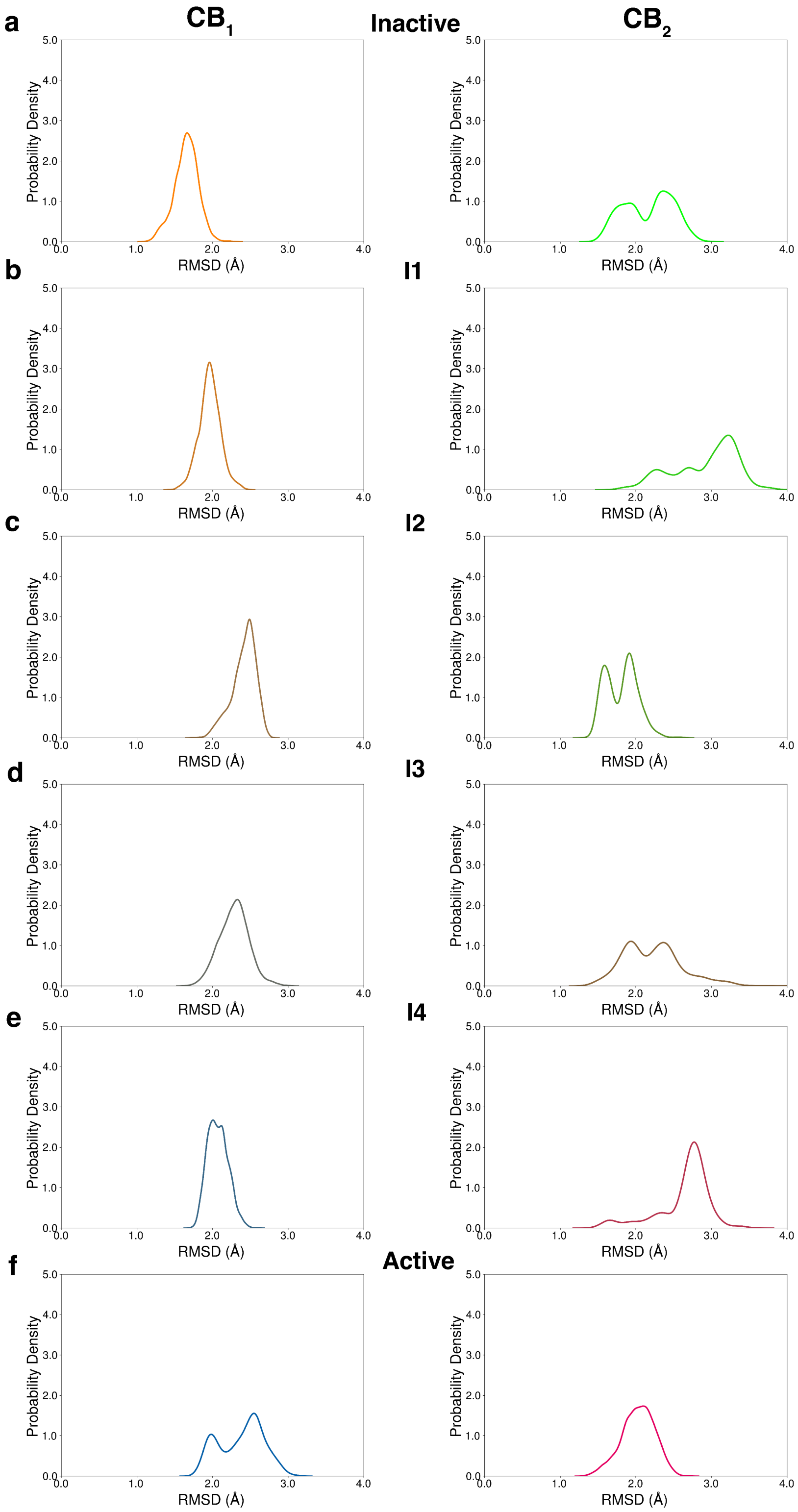

Supplementary Figure 18: RMSD calculations from the long timescale MD simulation performed on each metastable state (Inactive (**a**), I1 (**b**), I2 (**c**), I3 (**d**), I4 (**e**), Active (**f**)). Left panel represents CB_1 and right panel represents CB_2. Colors for CB_1 metastable states change from orange to blue gradually from inactive to active state. Colors for CB_2 metastable states change from green to purple gradually from inactive to active state. RMSD calculations were performed from the initial structure of simulation.

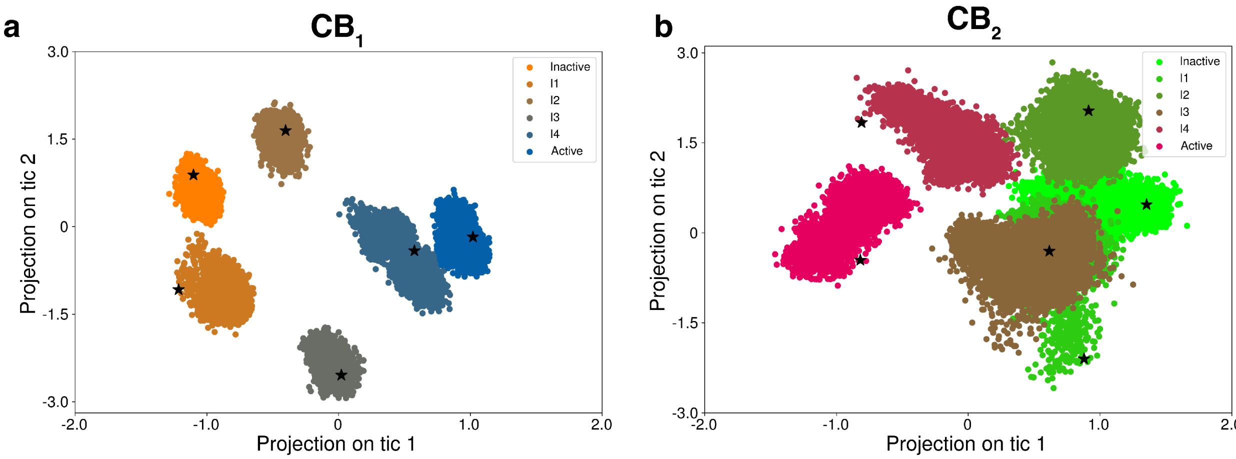

Supplementary Figure 19: Long time scale simulations are projected on tIC dimensions calculated from adaptive sampling data. tICs were estimated based on residue pair distances obtained from RRCS (described in the method section). Colors for CB_1 (**a**) metastable states change from orange to blue gradually from inactive to active state. Colors for CB_2 (**b**) metastable states change from green to purple gradually from inactive to active state. The star marker represents the starting point of the simulation for each state.

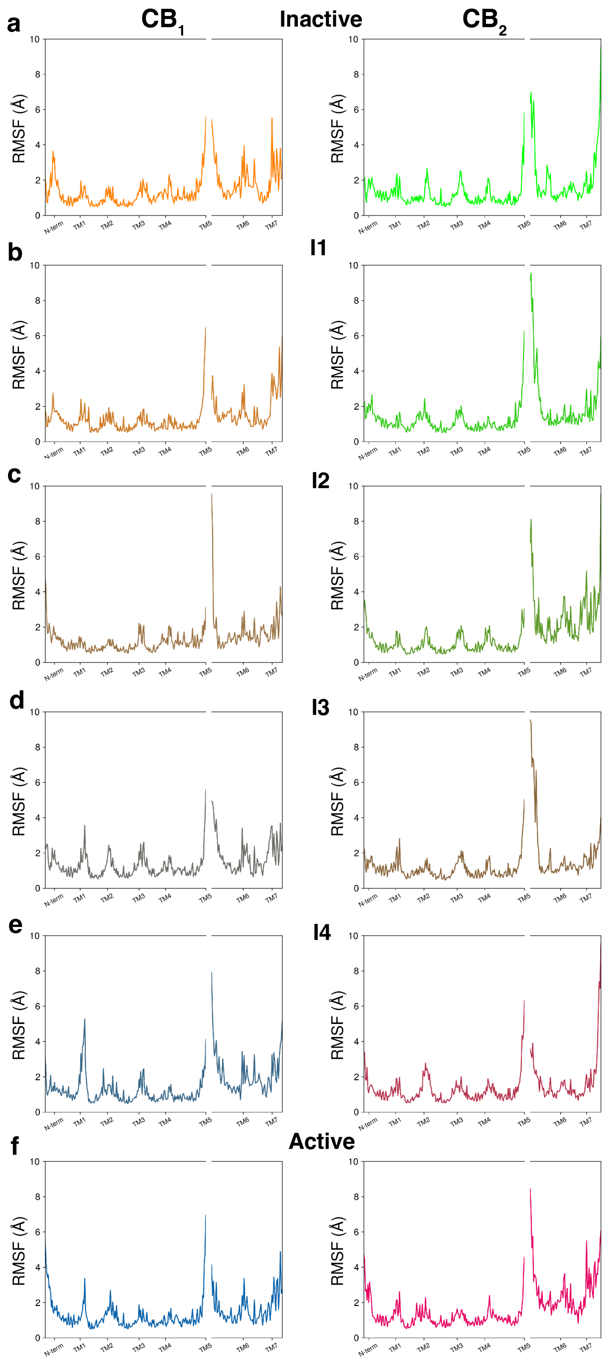

Supplementary Figure 20: RMSF calculations from the long timescale MD simulation performed on each macrostate (Inactive (**a**), I1 (**b**), I2 (**c**), I3 (**d**), I4 (**e**), Active (**f**)). Left panel represents CB_1 and right panel represents CB_2. Colors for CB_1 (**a**) metastable states change from orange to blue gradually from inactive to active state. Colors for CB_2 (**b**) metastable states change from green to purple gradually from inactive to active state. RMSF calculations were performed from the initial structure of simulation. Broken x-axis region represents the truncated ICL3 region.

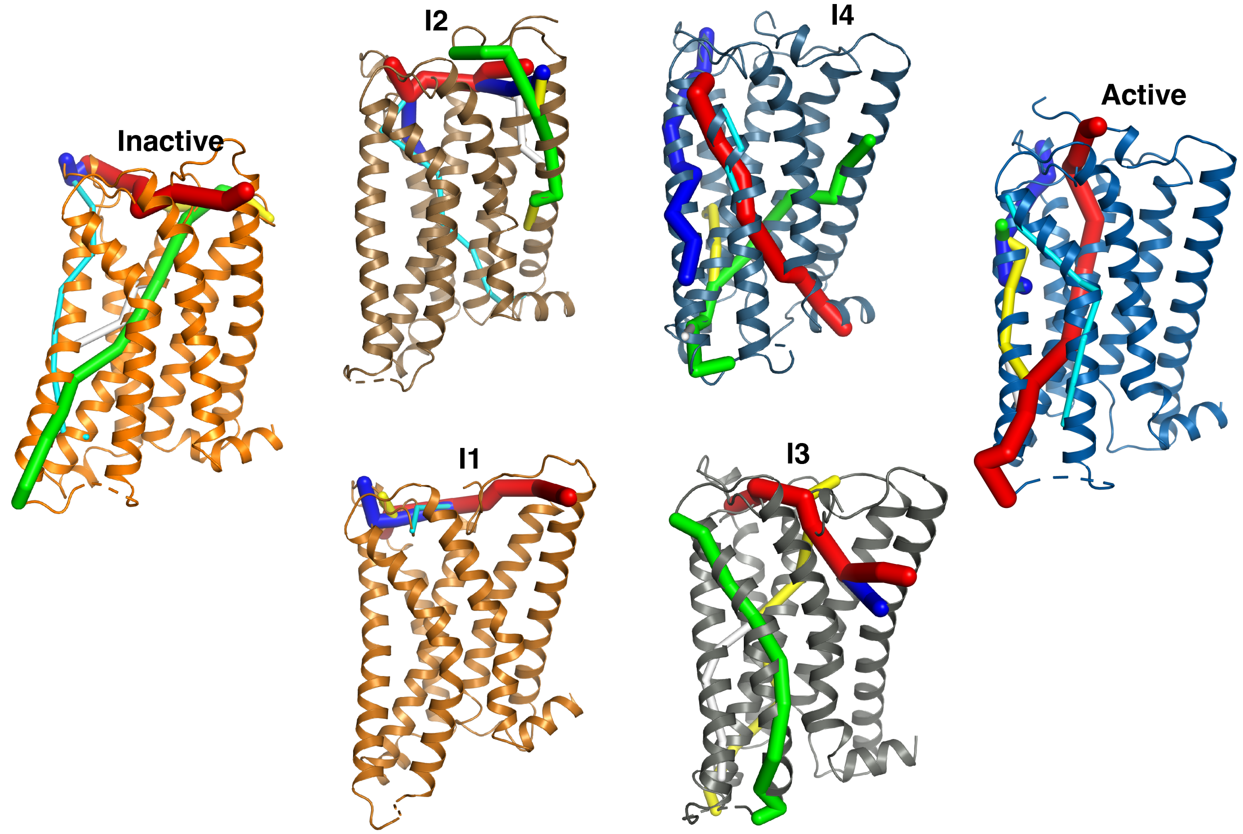

Supplementary Figure 21: Allosteric communication paths calculated for each metastable state for CB_1. Six tunnels with highest numbers of allosteric networks are shown. Tunnel radius are sorted (thicker to thinner) based on the number of allosteric networks in the tunnels.

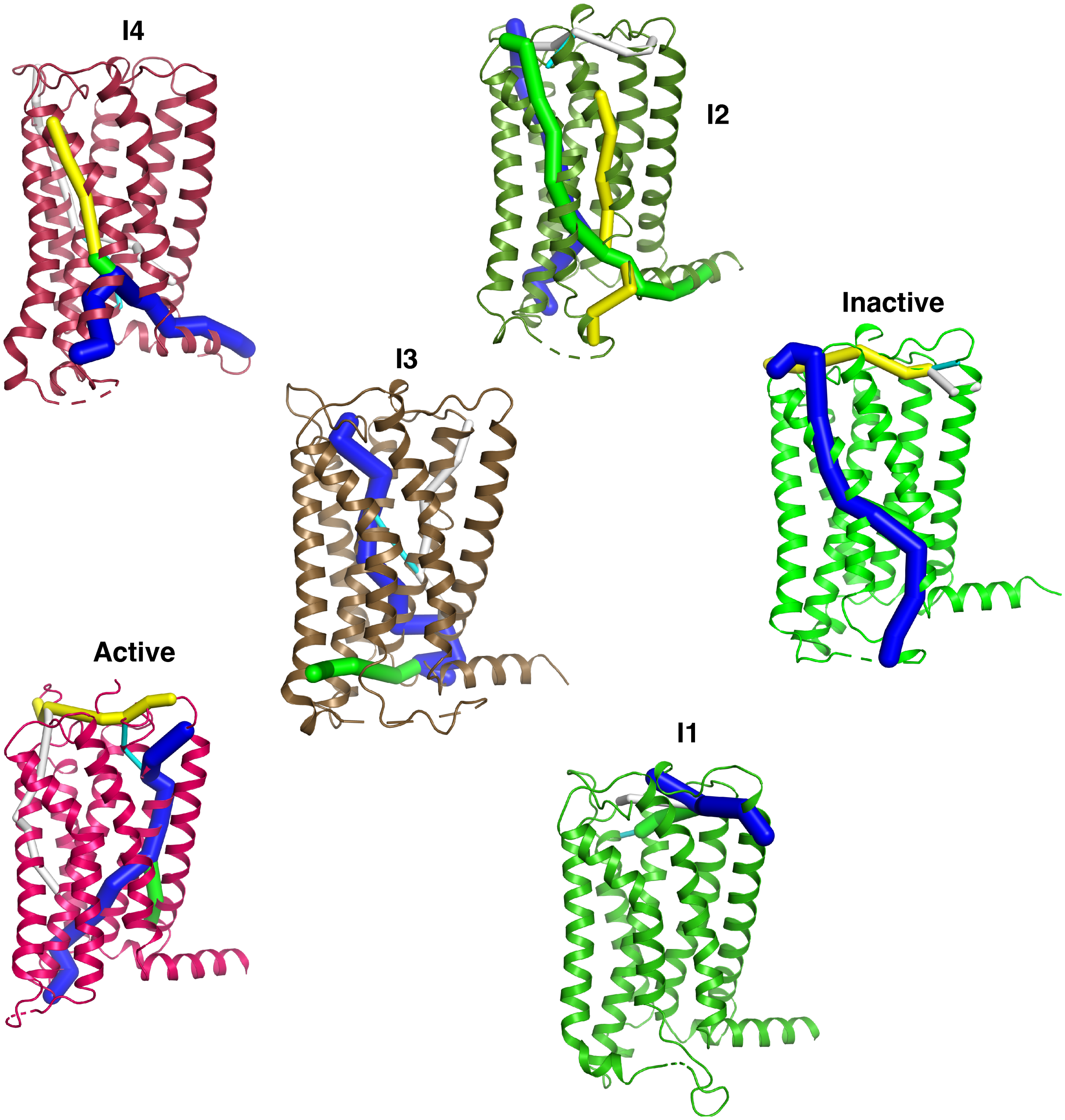

Supplementary Figure 22: Allosteric communication paths calculated for each metastable state for CB_2. Six tunnels with highest numbers of allosteric networks are shown. Tunnel radius are sorted (thicker to thinner) based on the number of allosteric networks in the tunnels.

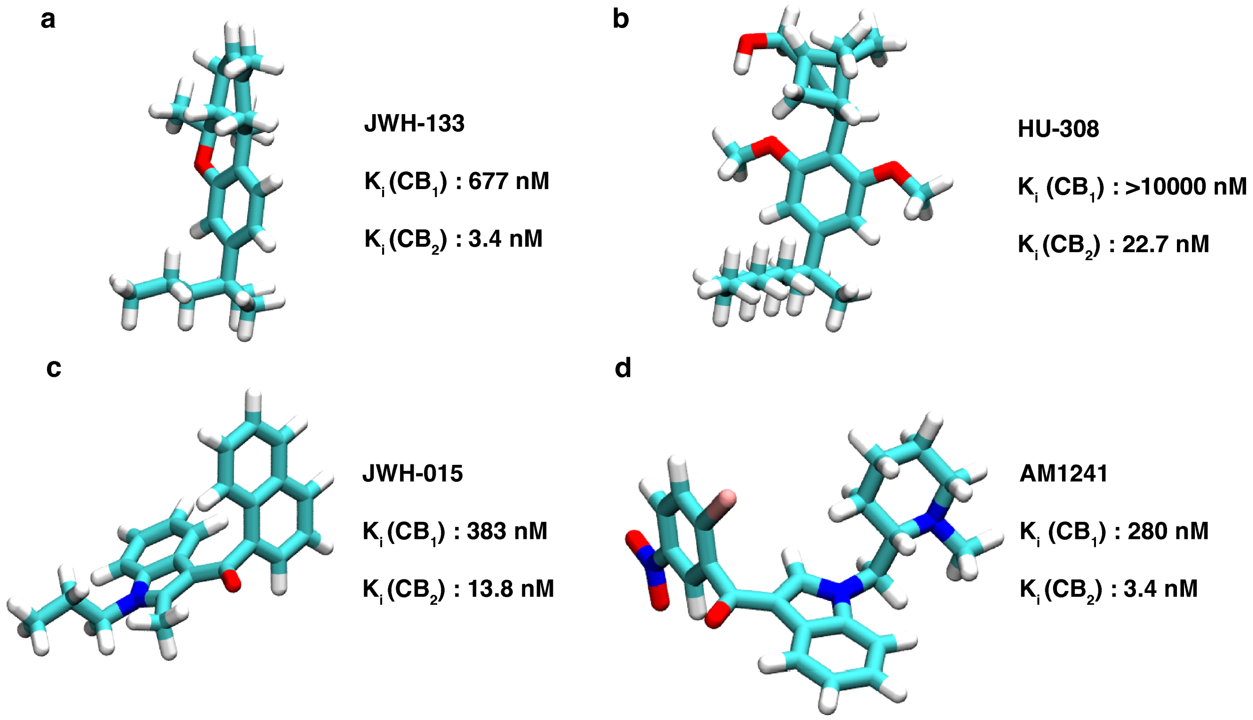

Supplementary Figure 23: CB_2 selective ligands (JWH-133 (**a**), HU-308 (**b**), JWH-015 (**c**), AM1241(**d**) considered for the docking study shown as sticks.

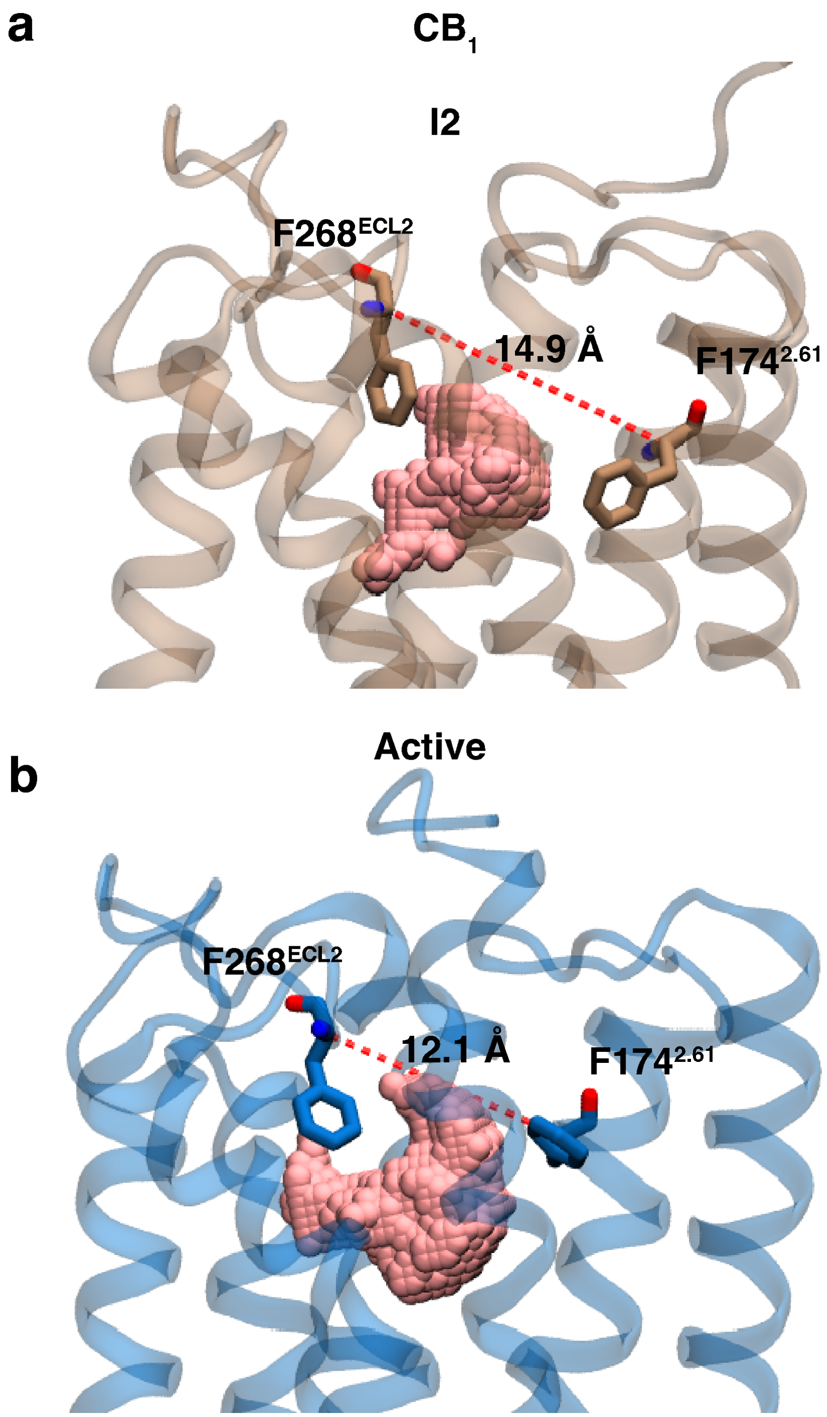

Supplementary Figure 24: Distance between F268^{ECL2} and F174$^{2.61}$ residues for I2 (**a**) and active (**b**) metastable states CB$_1$ are shown as red dotted lines. Proteins are shown as transparent cartoon. Pocket volume calculated using POVME software are shown as beads.

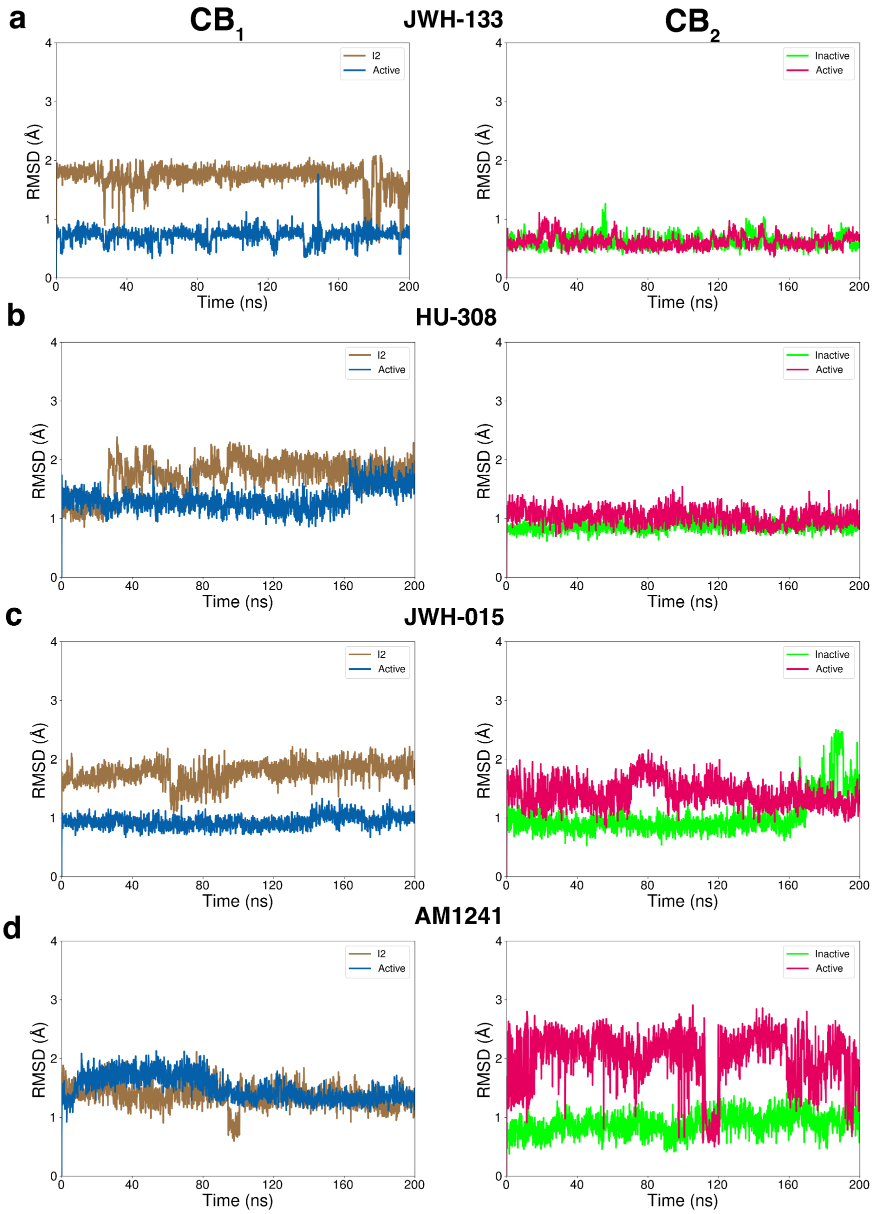

Supplementary Figure 25: Ligand RMSD calculations (JWH-133 (**a**), HU-308 (**b**), JWH-015 (**c**), AM1241(**d**) from the long timescale MD simulation performed on ligand bound CB_1 (intermediate state 2 (color: Brown) and active (color: Blue) state) and CB_2 (inactive (color: Green) and active states (color: Purple)) metastable states are plotted against time. Left panel represents CB_1 and right panel represents CB_2. RMSD calculations were performed from the initial structure of simulation.

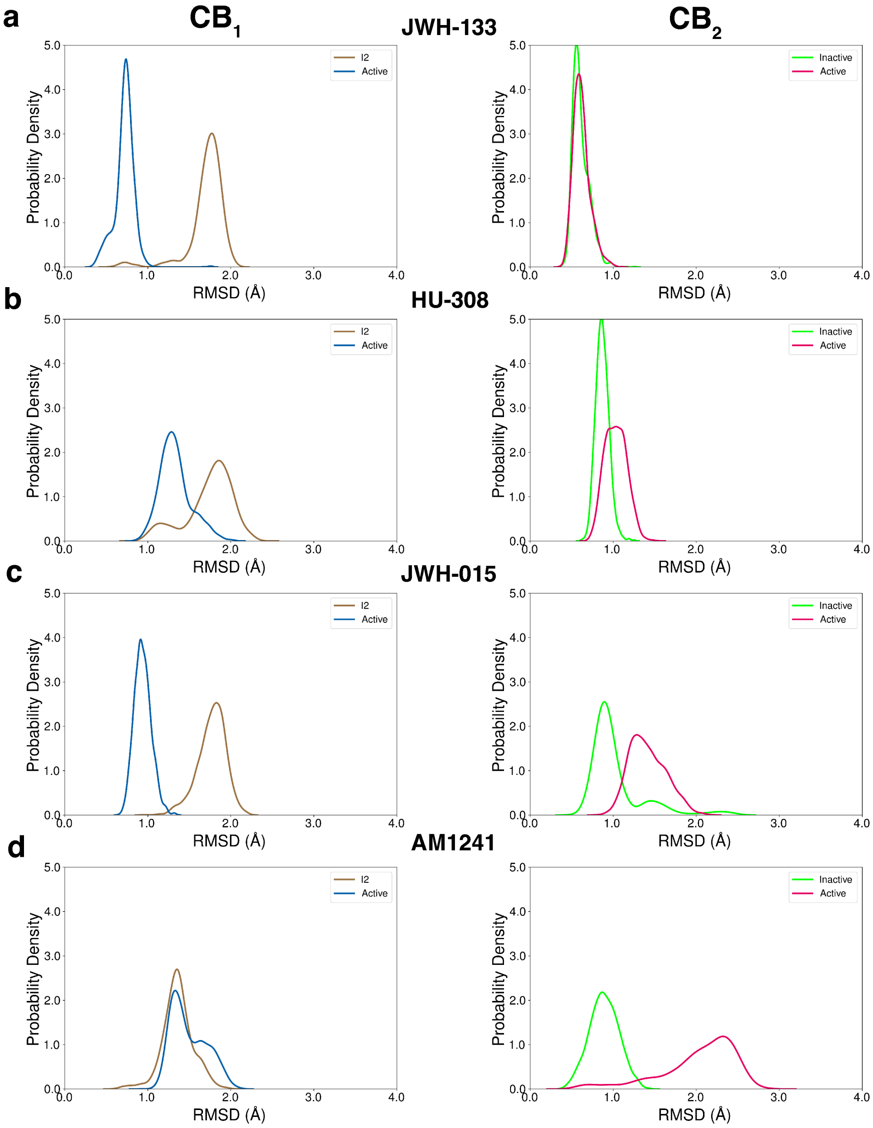

Supplementary Figure 26: Ligand RMSD calculations (JWH-133 (**a**), HU-308 (**b**), JWH-015 (**c**), AM1241(**d**) from the long timescale MD simulation performed on ligand bound CB_1(intermediate state 2 and active state) and CB_2 (inactive and active states) metastable states are plotted as a density plot. Left panel represents CB_1 and right panel represents CB_2. RMSD calculations were performed from the initial structure of simulation.

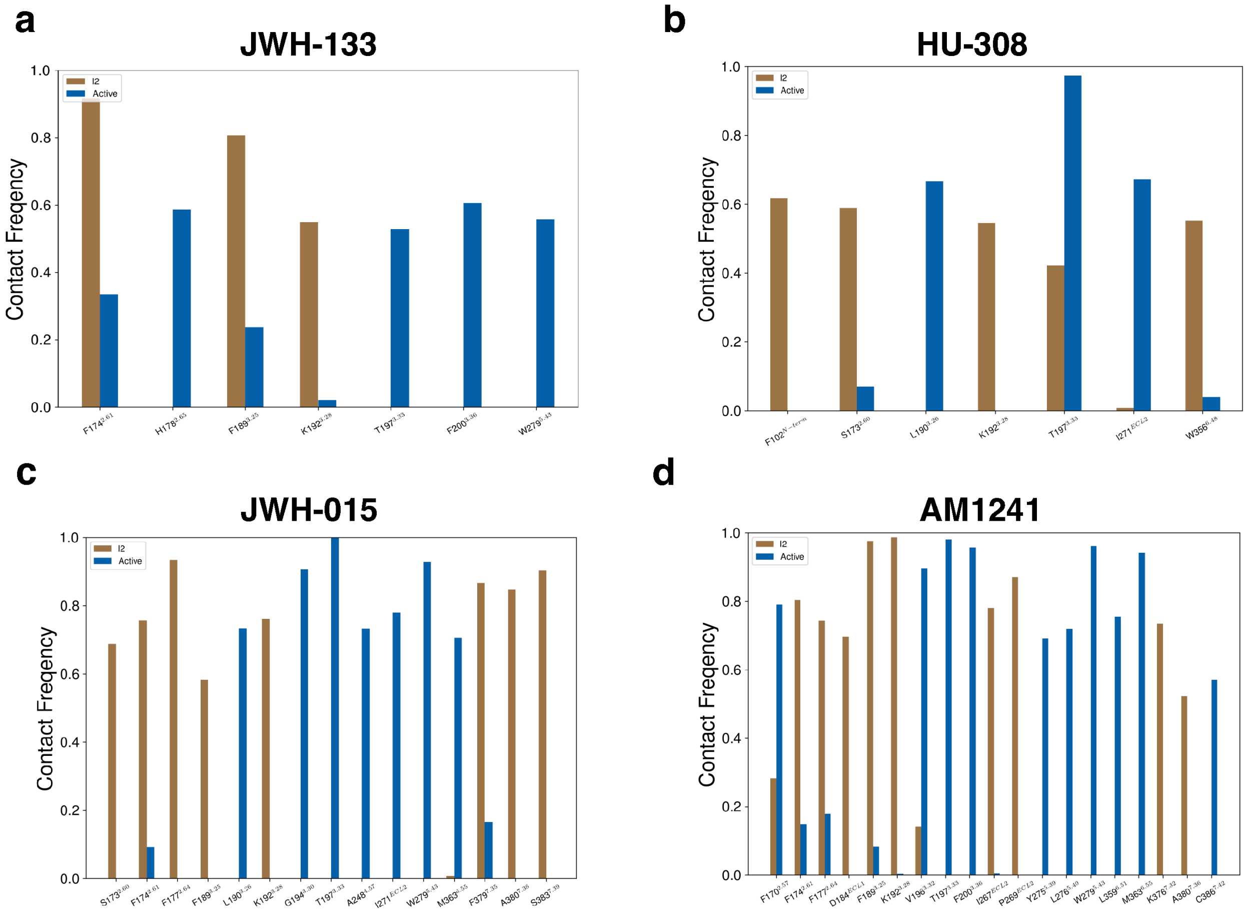

Supplementary Figure 27: Contact Frequencies of the residues that form stable contacts with the ligands (JWH-133 (**a**), HU-308 (**b**), JWH-015 (**c**), AM1241(**d**) with I2 and active states of the CB_1 are shown as bar plots. I2 and active metastable states are shown in brown and blue color, respectively. Stable contacts are defined as contacts with more than 50% contact frequencies.

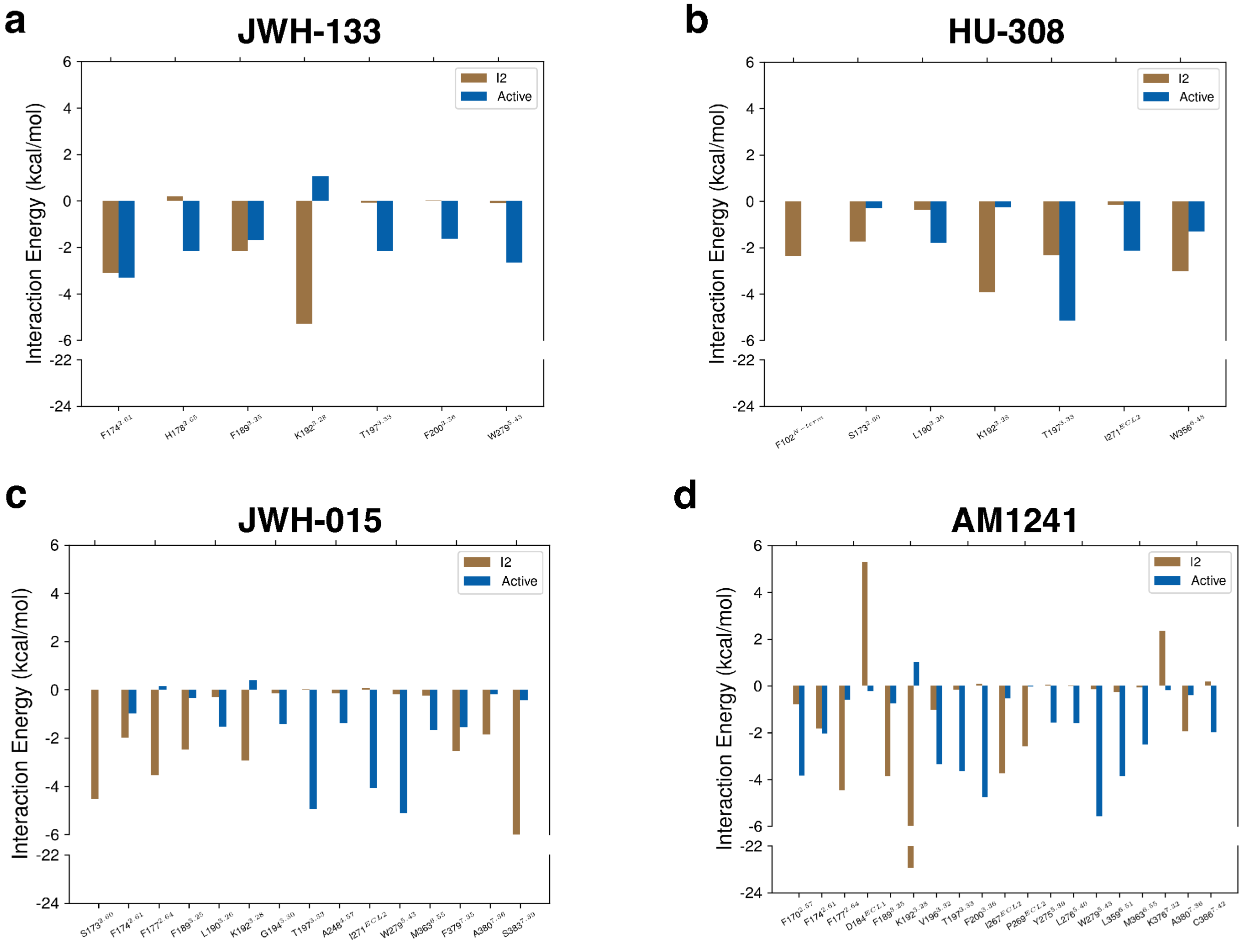

Supplementary Figure 28: Interaction energies (vdw + electrostatic) between the residues shown in Supplementary Figure 27 and ligands (JWH-133 (**a**), HU-308 (**b**), JWH-015 (**c**), AM1241(**d**) for both macrostates (I2 and active) of CB_1. I2 and active metastable states are shown in brown and blue color, respectively. Interaction energy calculations were performed with LIE method in Ambertools.

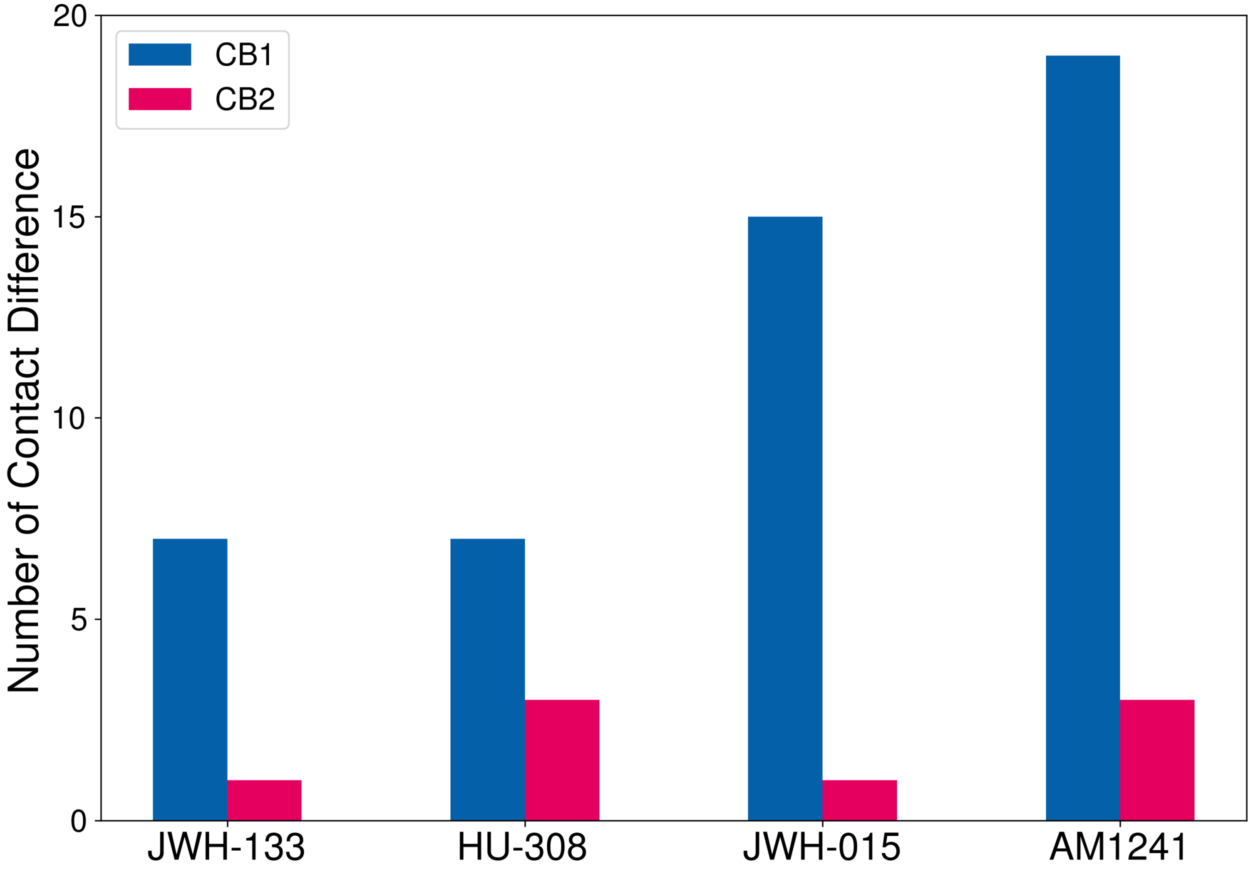

Supplementary Figure 29: Number of stable ligand and receptor contact differences between the two different macrostates of CB_1 (I2 and Active; color: Blue) and CB_2 (Inactive and active; color: Purple) are shown as Bar-plots. Blue bar represents CB_1, and pink bar represents CB_2.

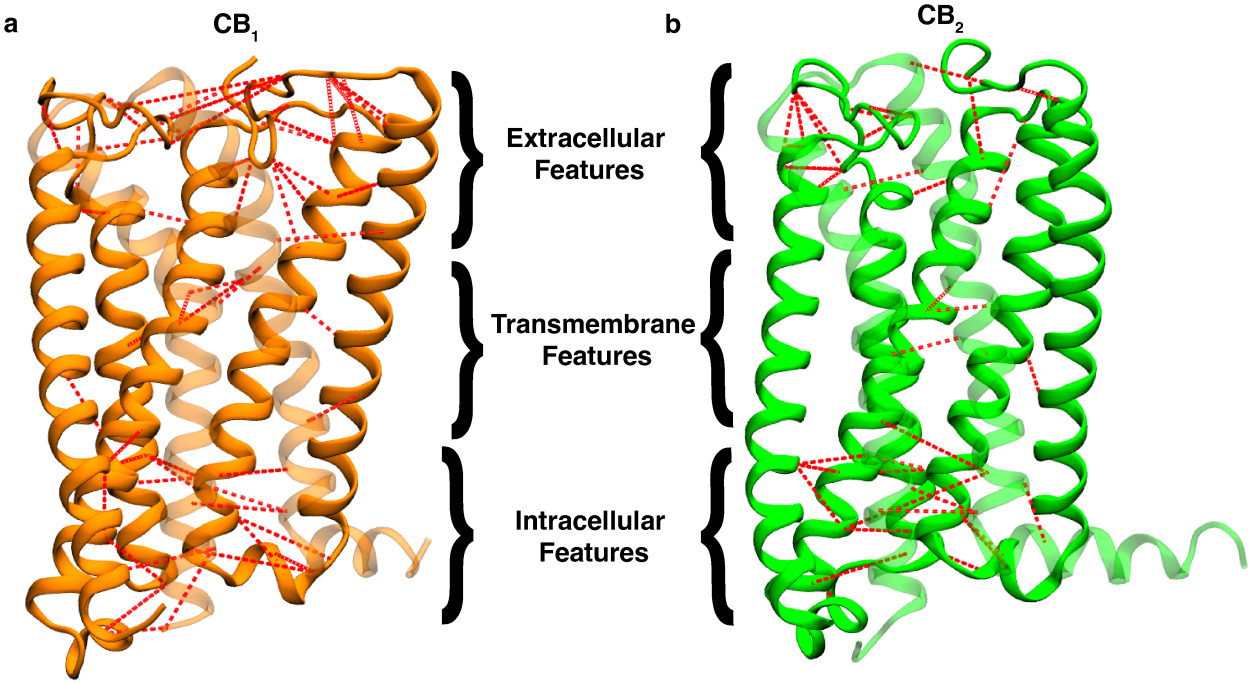

Supplementary Figure 30: Positions of the features calculated using RRCS analysis are shown as red dotted lines on cartoon representations of CB_1 inactive (color: Orange) (**a**) and CB_2 inactive (color: Green) (**b**) structures.

Supplementary Table 10: Distance and angle features used for adaptive sampling of CB_1.

Position	Number	Type	Feature
Extracellular helical movement	1	distance	$Q^{1.32}(C\alpha)$-$F^{3.25}(C\alpha)$
	2	distance	$Q^{1.32}(C\alpha)$-$P^{4.60}(C\alpha)$
	3	distance	$Q^{1.32}(C\alpha)$-$L^{5.40}(C\alpha)$
	4	distance	$Q^{1.32}(C\alpha)$-$D^{6.58}(C\alpha)$
	5	distance	$Q^{1.32}(C\alpha)$-$T^{7.33}(C\alpha)$
	6	distance	$D^{2.63}(C\alpha)$-$F^{3.25}(C\alpha)$
	7	distance	$D^{2.63}(C\alpha)$-$P^{4.60}(C\alpha)$
	8	distance	$D^{2.63}(C\alpha)$-$L^{5.40}(C\alpha)$
	9	distance	$D^{2.63}(C\alpha)$-$D^{6.58}(C\alpha)$
	10	distance	$D^{2.63}(C\alpha)$-$T^{7.33}(C\alpha)$
N-loop movement	11	distance	$M103^{N-term}(C\alpha)$-$D^{2.50}(C\alpha)$
	12	distance	$M103^{N-term}(C\alpha)$-$F268^{ECL2}(C\alpha)$
Toggle switch movement	13	Dihedral Angle (χ_2)	$F^{3.36}$
	14	Dihedral Angle (χ_2)	$W^{6.48}$
	15	distance	$W^{6.48}(N\epsilon)$-$D^{2.50}(C\alpha)$
	16	distance	$W^{6.48}(N\epsilon)$-$L^{5.50}(C\alpha)$
	17	distance	$F^{3.36}(C\gamma)$-$D^{2.50}(C\alpha)$
	18	distance	$F^{3.36}(C\gamma)$-$L^{5.50}(C\alpha)$
Intracellular helical movement	19	distance	$R^{3.50}(C\alpha)$-$K^{6.35}$ $(C\alpha)$
	20	distance	$Y^{2.40}(OH)$-$Y^{7.53}(OH)$
	21	distance	$I^{5.54}(C\alpha)$-$Y^{7.53}(OH)$
	22	distance	$A^{4.45}(C\alpha)$-$F^{2.42}(C\gamma)$
	23	Dihedral Angle (χ_2)	$F^{2.42}$
	24	Dihedral Angle (χ_2)	$F^{4.46}$

Supplementary Table 11: Distance and angle features used for adaptive sampling of CB_2.

Position	Number	Type	Feature
Extracellular helical movement	1	distance	$Q^{1.31}(C\alpha)$-$F^{3.25}(C\alpha)$
	2	distance	$Q^{1.31}(C\alpha)$-$P^{4.60}(C\alpha)$
	3	distance	$Q^{1.31}(C\alpha)$-$L^{5.40}(C\alpha)$
	4	distance	$Q^{1.31}(C\alpha)$-$S^{6.58}(C\alpha)$
	5	distance	$Q^{1.31}(C\alpha)$-$K^{7.33}(C\alpha)$
	6	distance	$N^{2.63}(C\alpha)$-$F^{3.25}(C\alpha)$
	7	distance	$N^{2.63}(C\alpha)$-$P^{4.60}(C\alpha)$
	8	distance	$N^{2.63}(C\alpha)$-$L^{5.40}(C\alpha)$
	9	distance	$N^{2.63}(C\alpha)$-$D^{6.58}(C\alpha)$
	10	distance	$N^{2.63}(C\alpha)$-$K^{7.33}(C\alpha)$
N-loop movement	11	distance	$M26^{N-term}(C\alpha)$-$D^{2.50}(C\alpha)$
	12	distance	$M26^{N-term}(C\alpha)$-$F183^{ECL2}(C\alpha)$
Toggle switch movement	13	Dihedral Angle (χ_2)	$W^{6.48}$
	14	distance	$W^{6.48}(N\epsilon)$-$D^{2.50}(C\alpha)$
	15	distance	$W^{6.48}(N\epsilon)$-$L^{5.50}(C\alpha)$
	16	distance	$F^{3.36}(C\gamma)$-$D^{2.50}(C\alpha)$
	17	distance	$F^{3.36}(C\gamma)$-$L^{5.50}(C\alpha)$
Intracellular helical movement	18	distance	$R^{3.50}(C\alpha)$-$K^{6.35}$ $(C\alpha)$
	19	distance	$Y^{2.40}(OH)$-$Y^{7.53}(OH)$
	20	distance	$I^{5.54}(C\alpha)$-$Y^{7.53}(OH)$

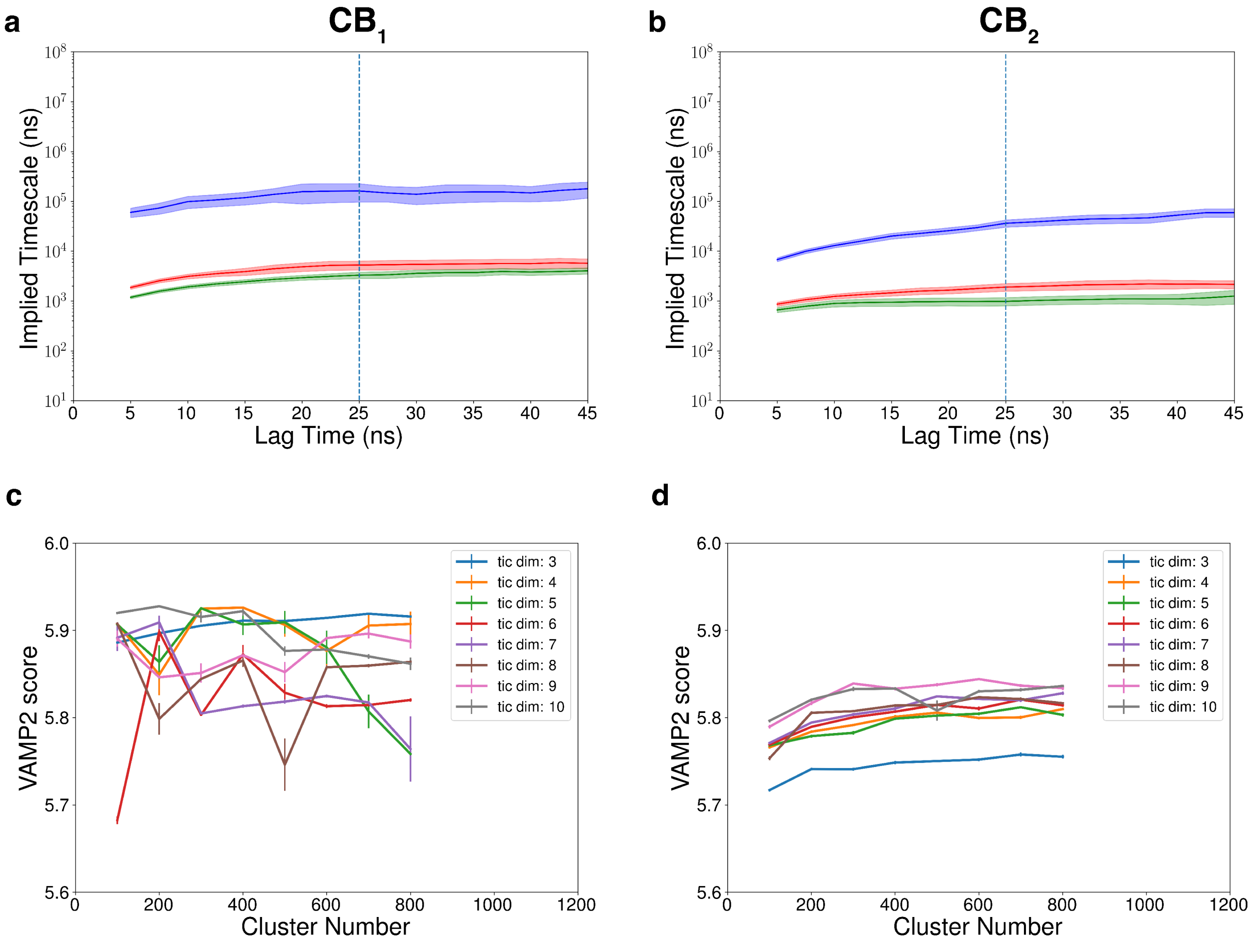

Supplementary Figure 31: First three implied timescale obtained from MSM is plotted against lag time for CB_1 (**a**) and CB_2 (**b**). Error bars are calculated using bootstrapping method by building 10 different samples with 80% of the total number of trajectories. 25 ns was selected as lagtime to build final MSM. VAMP-2 score was plotted against cluster numbers for CB_1 (**c**) and CB_2 (**d**). These calculations were performed for different tIC-dimensions. Error bars were calculated by tenfold cross validation.

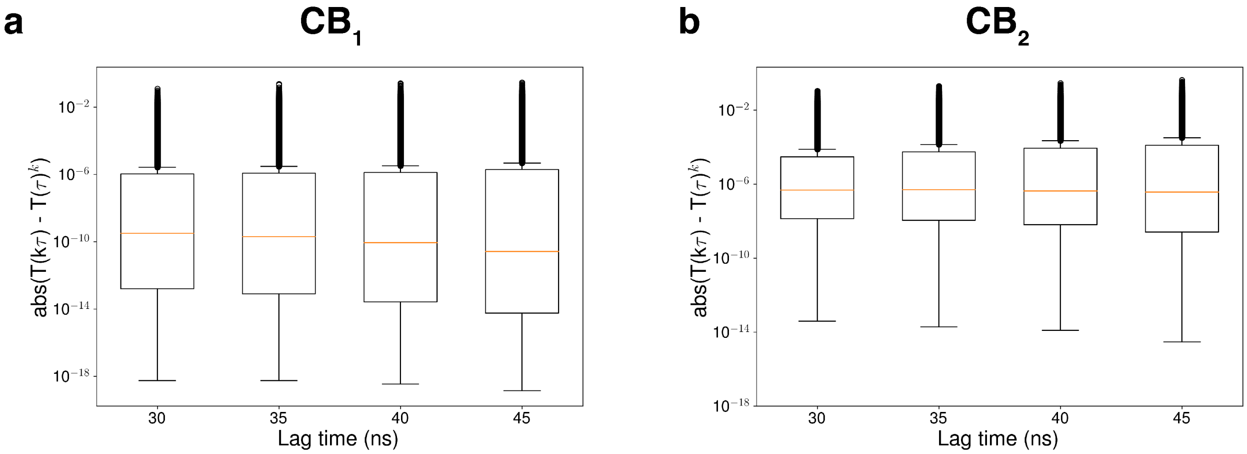

Supplementary Figure 32: Absolute difference between the elements of $T(k\tau)$ and $[T(\tau)]^k$ are shown with respected different k values for CB_1 (**a**) and CB_2 (**b**).

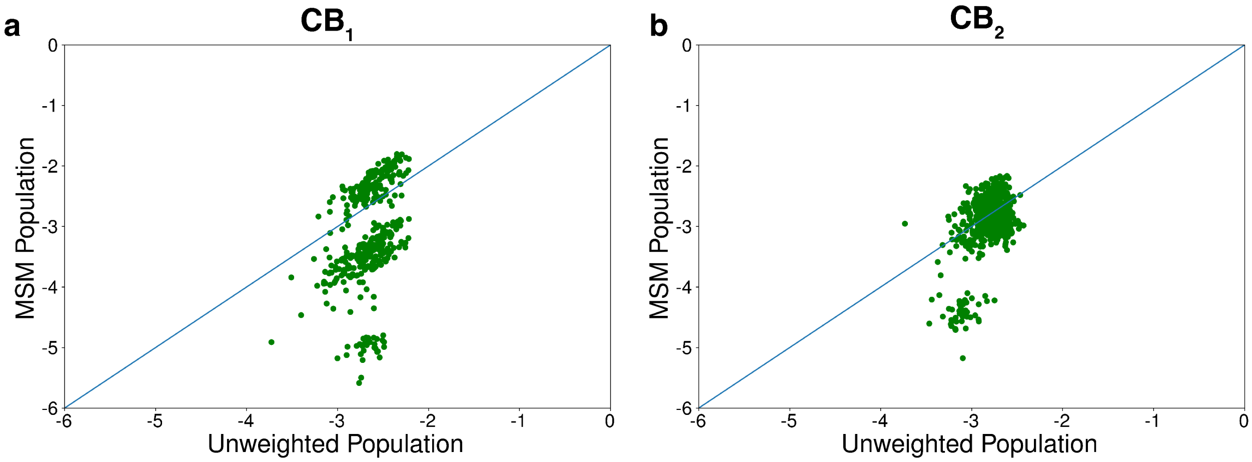

Supplementary Figure 33: Probability of population of each cluster obtained from adaptive sampling are plotted against the MSM reweighted probability for CB_1 (**a**) and CB_2 (**b**). Each dot represents each cluster. Probabilities are plotted in log scale.

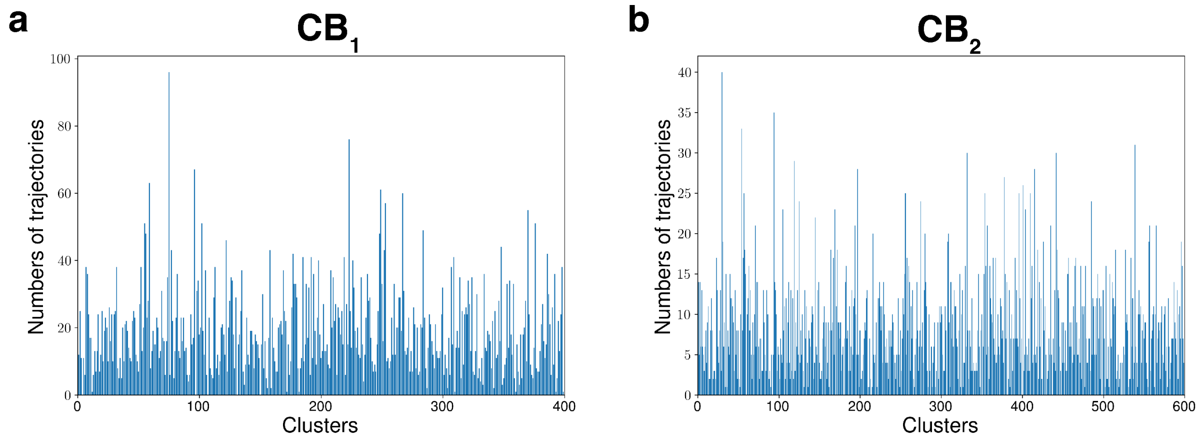

Supplementary Figure 34: Bar plots in (**a**) and (**b**) show number of simulation trajectories from each cluster of CB_1 (A) and CB_2(B) conformational ensemble. Each bar represents one cluster.

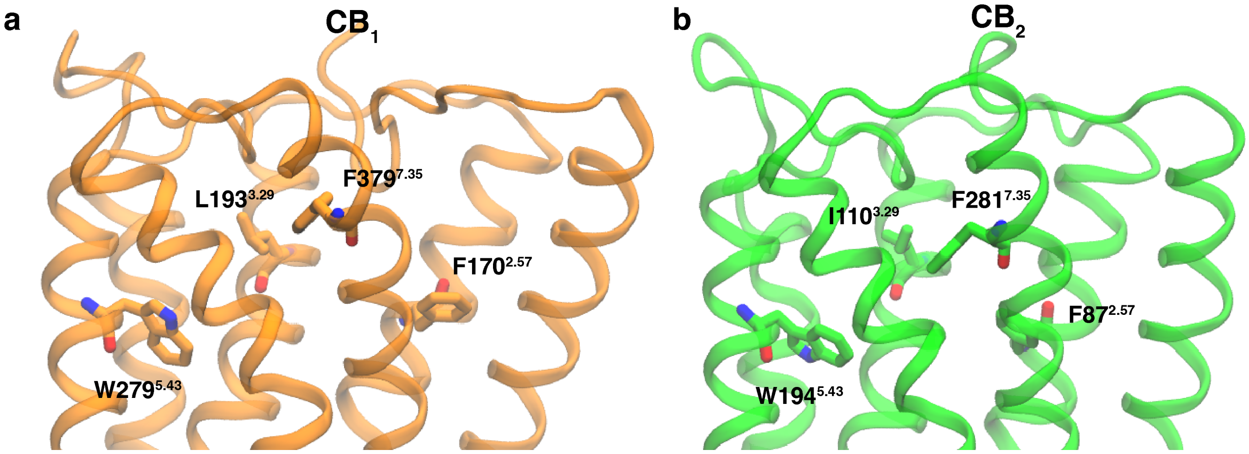

Supplementary Figure 35: Cartoon representation of CB_1 (**a**) and CB_2 (**b**) showing the binding pocket residues as sticks. Center of mass of the α-carbon of these residues were considered as docking center for docking calculation.

References

(1) Kohlhoff, K. J. *et al.* Cloud-based simulations on google exacycle reveal ligand modulation of GPCR activation pathways. *Nature Chemistry* **6**, 15–21 (2013).

(2) Dutta, S., Selvam, B., Das, A. & Shukla, D. Mechanistic origin of partial agonism of tetrahydrocannabinol for cannabinoid receptors. *Journal of Biological Chemistry* **298(4)**, 101764 (2022).

(3) Hua, T. *et al.* Crystal structure of the human cannabinoid receptor CB1. *Cell* **167**, 750–762.e14 (2016).

(4) Hua, T. *et al.* Crystal structures of agonist-bound human cannabinoid receptor CB1. *Nature* **547**, 468–471 (2017).

(5) Eddy, M. T., Martin, B. T. & Wüthrich, K. A2a adenosine receptor partial agonism related to structural rearrangements in an activation microswitch. *Structure* **29**, 170–176.e3 (2021).

(6) Hua, T. *et al.* Activation and signaling mechanism revealed by cannabinoid receptor-gi complex structures. *Cell* **180**, 655–665.e18 (2020).

(7) Rosenbaum, D. M., Rasmussen, S. G. F. & Kobilka, B. K. The structure and function of g-protein-coupled receptors. *Nature* **459**, 356–363 (2009).

(8) Latorraca, N. R., Venkatakrishnan, A. J. & Dror, R. O. GPCR dynamics: Structures in motion. *Chemical Reviews* **117**, 139–155 (2016).

(9) Weis, W. I. & Kobilka, B. K. The molecular basis of g protein–coupled receptor activation. *Annual Review of Biochemistry* **87**, 897–919 (2018).

(10) Shao, Z. *et al.* Structure of an allosteric modulator bound to the CB1 cannabinoid receptor. *Nature Chemical Biology* **15**, 1199–1205 (2019).

(11) Kumar, K. K. *et al.* Structure of a signaling cannabinoid receptor 1-g protein complex. *Cell* **176**, 448–458.e12 (2019).

(12) Shao, Z. *et al.* High-resolution crystal structure of the human CB1 cannabinoid receptor. *Nature* **540**, 602–606 (2016).

(13) Wang, X. *et al.* A genetically encoded f-19 NMR probe reveals the allosteric modulation mechanism of cannabinoid receptor 1. *Journal of the American Chemical Society* **143**, 16320–16325 (2021).

(14) Li, X. *et al.* Crystal structure of the human cannabinoid receptor CB2. *Cell* **176**, 459–467.e13 (2019).

(15) Xing, C. *et al.* Cryo-EM structure of the human cannabinoid receptor CB2-gi signaling complex. *Cell* **180**, 645–654.e13 (2020).

(16) Roe, D. R. & Cheatham III, T. E. Ptraj and cpptraj: software for processing and analysis of molecular dynamics trajectory data. *Journal of chemical theory and computation* **9**, 3084–3095 (2013).

(17) McGibbon, R. T. *et al.* Mdtraj: A modern open library for the analysis of molecular dynamics trajectories. *Biophysical Journal* **109**, 1528 – 1532 (2015).

(18) Wang, J., Wolf, R. M., Caldwell, J. W., Kollman, P. A. & Case, D. A. Development and testing of a general amber force field. *Journal of computational chemistry* **25**, 1157–1174 (2004).

(19) Wang, J., Wang, W., Kollman, P. A. & Case, D. A. Automatic atom type and bond type perception in molecular mechanical calculations. *Journal of molecular graphics and modelling* **25**, 247–260 (2006).

(20) de Amorim, H. L., Caceres, R. & Netz, P. Linear interaction energy (LIE) method in lead discovery and optimization. *Current Drug Targets* **9**, 1100–1105 (2008).
